# Spatiotemporal dynamics reveals forest rejuvenation, fragmentation, and edge effects in an Atlantic Forest hotspot, the Pernambuco Endemism Center, northeastern Brazil

**DOI:** 10.1371/journal.pone.0291234

**Published:** 2023-09-08

**Authors:** Thiago da Costa Dias, Luís Fábio Silveira, Mercival Roberto Francisco

**Affiliations:** 1 Programa de Pós-Graduação em Ecologia e Recursos Naturais, Universidade Federal de São Carlos, São Carlos, São Paulo, Brazil; 2 Seção de Aves, Museu de Zoologia da Universidade de São Paulo, São Paulo, Brazil; 3 Departamento de Ciências Ambientais, Universidade Federal de São Carlos, Campus de Sorocaba, Sorocaba, São Paulo, Brazil; Van Lang University: Truong Dai hoc Van Lang, VIET NAM

## Abstract

Large forested tracts are increasingly rare in the tropics, where conservation managers are often presented with the challenge of preserving biodiversity in small and isolated fragments. The Atlantic Forest is one of the world’s most important biodiversity hotspots, jeopardized by habitat loss and fragmentation. The Pernambuco Endemism Center (PEC) is the most degraded of the Atlantic Forest regions and because of the dramatic levels of deforestation, fragmentation, and ongoing species losses, studies on the distribution and configuration of the PEC’s forest cover are necessary. However, across dynamic tropical landscapes, investigating changes over time is essential because it may reveal trends in forest quality attributes. Here, we used Google Earth Engine to assess land use and land cover data from MapBiomas ranging from 1985 to 2020 to calculate current landscape metrics and to reveal for the first time the spatiotemporal dynamics of the PEC’s forests. We identified a forest cover area that ranged from 571,661 ha in 1985 to 539,877 ha in 2020, and about 90% of the fragments were smaller than 10 ha. The average fragment size was about 11 ha, and only four fragments had more than 5,000 ha. Deforestation was mostly concentrated in northern Alagoas, southern Pernambuco, and non-coastal Paraíba and Rio Grande do Norte. On average, borders represented 53.6% of the forests from 1985 to 2020, and younger forests covered 52.3% of the area in 2017, revealing a vegetation rejuvenation process 2.5 times higher than in total Atlantic Forest. In 2017, older forest cores in fragments larger than 1000 ha (i.e., higher-quality habitats) represented only 12% of the remaining forests. We recommend that the amount of forest cover alone may poorly assist conservation managers, and our results indicate that ensuring legal protection and increasing surveillance of the PEC’s few last higher-quality habitats is urgently needed.

## Introduction

Tropical forests harbor more than half of all known species and are among the most endangered ecosystems on Earth [1, 2], with deforestation rates reaching around 5.5 Mha/yr^-1^ [3]. Besides reducing critical habitats for many species, deforestation also increases fragmentation and edge effects through the conversion of larger blocks of forests into smaller ones [4, 5]. Because habitat loss and fragmentation are the leading causes of wildlife erosion and ecosystem functioning depletion worldwide [6, 7], there is a broad consensus that protecting large areas of natural vegetation is the best way to achieve conservation purposes [8, 9]. However, large forested tracts have become increasingly rare in tropical forest hotspots, where conservation managers are often presented with the challenge of preserving biodiversity in small and isolated fragments [8, 10, 11].

Assessing the spatial patterns and distribution of the fragments in highly degraded habitats is important for conservation planning because they can predict the extinction risks of biodiversity components [12, 13], and because they can indicate the best remnants for the creation of protected areas [14, 15]. However, in dynamic human-modified landscapes, the replacement of older forests by secondary and younger habitats has been recently proved to be a secretive temporal effect that can masquerade the loss of high-quality habitats [16]. In these circumstances, simple assessments of land cover patterns may provide only part of the information needed for conservation planning due to the lack of the temporal component [17]. Investigating landscape changes over time is essential because it can reveal trends in deforestation and forest regeneration rates, and because temporal changes in fragment attributes (e.g., isolation, area, age, and edge effects) influence biodiversity distribution [18].

The Brazilian Atlantic Forest is one of the world’s most important biodiversity hotspots and it has been through a history of intense degradation since the European settlement more than 500 years ago [19–21]. Currently, only about 11.26% of its original 150 Mha remains, almost entirely (80%) in fragments smaller than 50 ha [22]. Despite the continuous deforestation process, it was recently revealed that the amount of vegetation cover was relatively constant during the last 30 years (around 28 Mha) due to a hidden process of substitution of old forests by areas of secondary vegetation, with losses of older habitats ranging from 220,000 to 80,000 ha/year^-1^ from 2000 to 2015 [16].

The Pernambuco Endemism Center (hereafter PEC) formerly comprised a 4.4 Mha area located north of the São Francisco River [23, 24], in northeastern Brazil [22]. Today, the PEC is the most degraded of the Atlantic Forest regions [22], challenging conservation managers due to the high concentration of threatened endemic taxa [25–28], the reason why it has been considered a hotspot within a hotspot [29]. About 360,455 ha of native forests were detected in the PEC using data from 2005, with about 60% of the total cover influenced by edge effects and the absence of fragments larger than 10,000 ha [22]. Another estimate carried out with data from 2001 to 2007 revealed 322,372 ha of forests, distributed into fragments smaller than 10 ha (38,504 ha), larger than 10 ha (121,081 ha), larger than 100 ha (114,440 ha), and larger than 1,000 ha (48,347 ha) [29].

Despite the efforts to characterize the distribution of the PEC’s habitats during the last decades [19, 22, 29, 30], previous studies did not capture the temporal dynamics of its forest cover. The variation in methodological procedures, associated with the fact that the last comprehensive study was conducted using data from 2007, has impeded proper interpretations about the temporal changes that occurred in the PEC’s forest cover, and there is a lack of information on the recent status of its habitats. Additionally, regional assessments on parameters related to habitat quality (e.g., vegetation age) over the Atlantic Forest are urgently needed for landscape management [16].

Here, we aimed to reveal the spatiotemporal dynamics of the most endangered habitat in the Americas during the last decades, providing an updated descriptive assessment of the changes that occurred in the configuration and distribution of the PEC’s habitats, as well as inferring the current state of its forests. We used Google Earth Engine (GEE) [31] to process MapBiomas annual land use and land cover layers [32] from 1985 to 2020 [32] and we calculated metrics related to the (i) amount of forest cover, (ii) number of fragments, (iii) average fragment size, (iv) largest fragments, (v) deforestation rates, (vi) forest regeneration rates, (vii) amount of core and edge areas, and the (vii) amount of older and younger forests. We predicted that the forest rejuvenation dynamic reported for the whole Atlantic Forest [16] could be even more remarkable in the PEC because differently from southern Atlantic Forest regions, large and well-preserved forest tracts no longer exist northern from the São Francisco River [22]. In this study, we incorporate forest quality parameters not previously considered by conservation managers and we developed the open-access GEE application “Forests of the PEC” (https://dias93thiago.users.earthengine.app/view/forests-of-the-pec) that may potentially assist conservation planning in this important biodiversity hotspot.

## Methods

### Description of the study area

**Fig 1:**
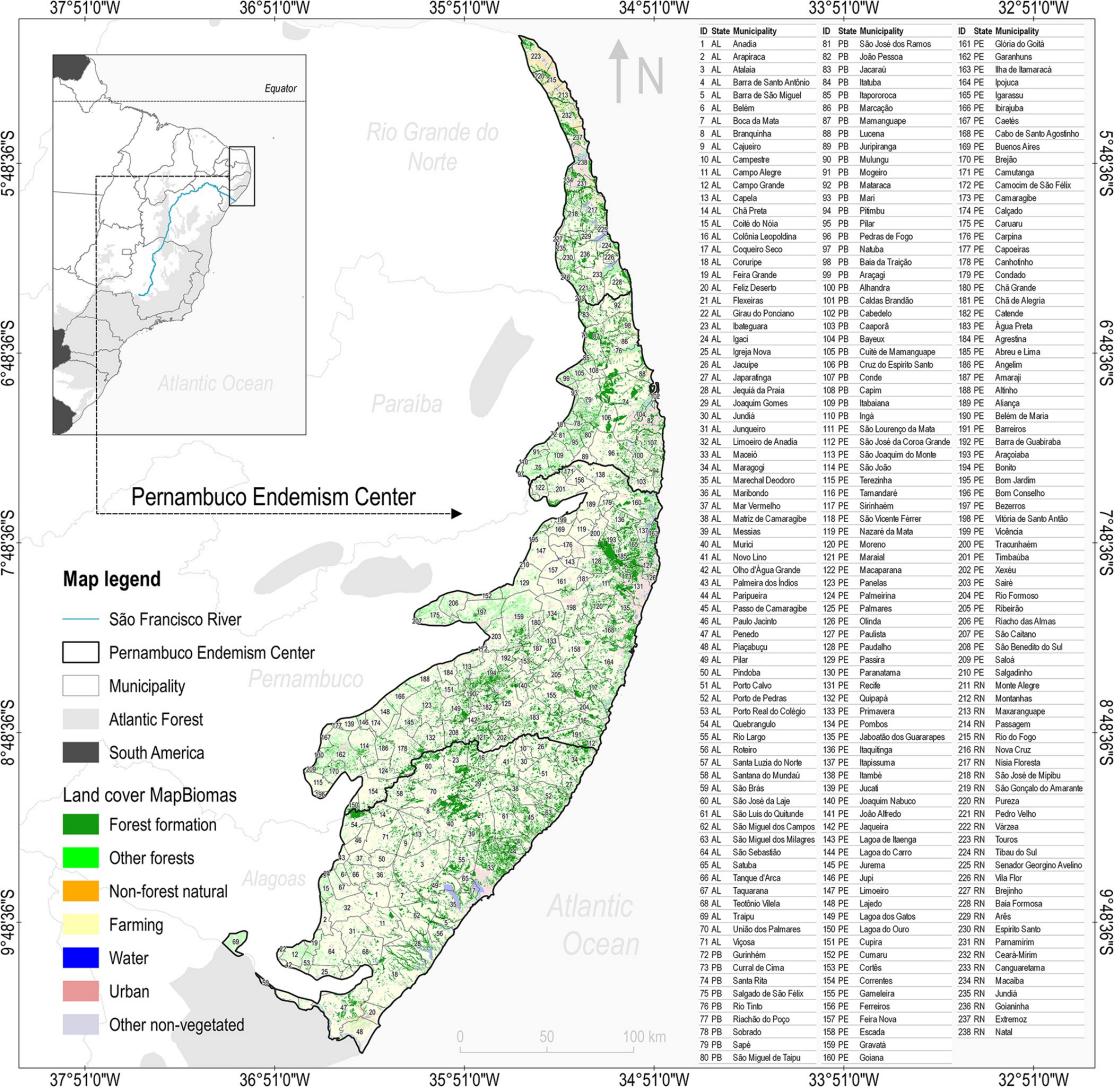
Location of the Pernambuco Endemism Center in the Brazilian Atlantic Forest of northeastern Brazil. The 2020 land use and land cover layer from MapBiomas Project [32] was adapted to show relevant aggregations of land cover classes. The class “forest formation” from annual layers represents a proxy of the PEC forest cover. The table on the right shows the identification (ID) and name of its municipalities. The additional layer representing administrative boundaries used in this map was obtained from the freely-available dataset provided by the Brazilian Institute of Geography and Statistics (IBGE).

The PEC is a biogeographic zone of the Brazilian Atlantic Forest located in northeastern Brazil across the states of Alagoas (AL), Pernambuco (PE), Paraíba (PB), and Rio Grande do Norte (RN) (Fig 1). Human interferences were responsible for the intense degradation of its forest formations since the 16th century, and the highest levels of fragmentation and deforestation occurred during the 1970s, mainly due to the activities of the sugar cane industry [33]. This intensive degradation reduced the forest cover to no more than 11.5% of its original size, mostly unprotected (protected areas account for about 1% of the forest cover) [22]. The PEC is located in a portion of the tropical zone where the predominant climate is Köppen’s As (tropical with a dry season), and smaller portions of the climate are Köppen’s Af (tropical without a dry season) are also found. Annual rainfall ranges from 1900–2200 mm in the coastal regions and 700–1000 mm in the western border, with annual mean temperature varying from 24 to 26° C [34]. The PEC went through four major waves of deforestation, the first three occurred between the sixteenth and eighteenth centuries, and the last started after 1975, during the establishment of the Proálcool Program (Brazilian Alcohol Program) [23]. The PEC is home to the highest number of globally threatened species in the Americas [26, 28, 35, 36]. Large mammals are currently extinct in the PEC (e.g., Puma *Puma concolor*, Jaguar *Panthera onca*, White-lipped Peccary *Tayassu pecari*, Brazilian Tapir *Tapirus terrestris*, Giant Ant-eater *Myrmecophaga tridactyla*, Gray Brocket *Mazama gouazoubira*), and at least half of its medium-sized mammals have disappeared over the last 500 years [29, 37, 38]. It was also at the PEC that the modern Brazilian bird extinctions were registered [26, 39, 40].

### Dataset

We used GEE to assess data from the sixth collection of the open-source MapBiomas Project, a collaborative initiative that provides annual (1985–2020) land use and land cover information at 30 m spatial resolution for Brazil using Landsat imagery [32]. In GEE, we retained only areas classified as forest formations, representing proxies of the Brazilian Atlantic Forest cover of the PEC. In the MapBiomas Project, all data was processed with methods that accounted for interferences from clouds or atmospheric haze, and several spatial and temporal post-classification filters were applied to ensure data quality [32]. According to MapBiomas, the Collection 6 Level 2 land use and land cover classes of the Atlantic Forest biome has a global accuracy of 85.5%, allocation disagreement of 8.3%, and area disagreement of 6.2% [32]. For the ‘forest formation’ class in Atlantic Forest biome (used in this study), overall accuracy ranged from 85.6% to 87.89% between 1985 e 2018 [32]. A stratified sample design that took the probabilities of sample weight adjustment into account was used by the MapBiomas team (three experts) to validate data using more than 12,000 points [32]. Following the minimum forest size (0.5 ha) established by FAO’s Global Forest Resource Assessment [41], we removed all fragments with less than six pixels (0.54 ha) using Rook’s case for pixel adjacency [42]. Using the ‘clumpSize’ function in ‘bfastSpatial’ package [43] in R [44], we detected clumps of connected cells, assigned unique identification numbers (IDs) for each clump (forest fragment), and calculate their sizes based on the number of connected pixels, which were lastly converted to hectares [43]. For temporal analyses, we selected a time threshold of 35 years (1985–2020) due to the availability of the annual data from MapBiomas, which is the open-access data source with the highest temporal resolution for Brazil [43]. All layers resulting from this study are freely available in the GEE application “Forests of the PEC”, and can be downloaded by assessing the following link https://dias93thiago.users.earthengine.app/view/forests-of-the-pec. Additionally, all of the GEE and R scripts were provided in Supplementary Material.

### Forest cover, number of fragments, average, and largest fragment sizes

In GEE, we evaluated temporal changes in the forest cover extent and composition by calculating the overall forest area and the areas of fragments > 10 ha (very small), 10–100 ha (small), 100–1,000 ha (medium), and > 1,000 ha (large). For comparative purposes, we followed the fragment size classes used in previous works on the PEC [29]. We also calculated the total number of fragments, and the number of fragments according to the abovementioned size classes. For forest area and number of fragments, we identified linear trends over time by applying a bottom-up breakpoint analysis using segmented package [45] in R. After selecting the number and location of breakpoints using Bayesian Information Criterion (BIC), this analysis subdivides a time series into phases with distinct trends and slopes [46]. We determined the average fragment size by dividing the total forest cover area by the number of fragments, and we inferred the size of PEC’s largest fragment. To assess the current distribution of forests for each municipality of the PEC in 2020, we generated layers representing the forest cover area (total) and the number of fragments (very small, small, medium, and large fragments). We chose to calculate metrics for each municipality to facilitate the interpretation of our results by state government environmental agencies and policy makers. To capture processes that may operate in different spatial scales, we developed a tool in the GEE application “Forests of the PEC” which permits regions of interest to be defined by users. We additionally identified the remaining largest fragments of the PEC (fragments larger than 5,000 ha) in 2020 and we generated information on their core and edge areas, as well as the areas composed by older and younger forests (see sections below).

### Deforestation, forest regeneration, and identification of older and younger forests

We assessed the current amount and the distribution of older (> 35 years) and younger (< 35 years) forests of the PEC in 2017 using GEE. We used the pixels classified as forests in 1985 with no event of deforestation registered until 2017 as proxies of the older forests of the PEC (see methodology for deforestation classification below). Pixels classified as forests in 2017 that did not match these conditions were classified as younger forests. We generated layers containing the overall current distribution of older and younger forests, and their current distributions per municipality. To measure deforestation and forest regeneration from 1987 to 2017, we implemented a moving window-based temporal filter in GEE [16, 47].

Year-specific deforestation events were assumed when a given pixel was classified as forest for the two previous years (t– 2, t– 1) and as non-forest in the current (t) and subsequent year (t + 1) (S1 Fig) [16]. We classified year-specific forest regeneration events when pixels were classified as non-forest for the two previous years (t– 2, t– 1), and then as forest in the current year (t) and in the next three subsequent years (t + 1, t + 2, t + 3) (S2 Fig) [16]. Moreover, we investigated deforestation events across a range of fragment sizes (S3 Fig). Using the annual deforestation layers, we extracted and averaged the fragment size during the two previous years (t– 2, t– 1) for each deforestation pixel. We then classified the resulting pixels of deforestation into deforestation of very small, small, medium, and large fragments.

We discriminated deforestation of older forests by calculating the year of the first deforestation event in pixels classified as forests in 1985. We only classified the first deforestation event of a given 1985 forest pixel as deforestation of older forests. For each year, we remapped the values of deforestation from one to the value corresponding to its year of occurrence. After that, for each pixel, we extracted the minimum value (corresponding to the first event of deforestation) and masked the new classified images to remove all pixels not classified as forests in 1985 (S4 Fig). We classified all the other deforestation events that did not matched the abovementioned conditions as deforestation of younger forests. We addressed deforestation rates of older forests only after 2000 because at least part of the deforestation that occurred in the first years of our study time may stand as deforestation of younger forests that grew just before 1985 [16]. To evaluate the participation of each municipality in forest loss and gain rates across the PEC, we additionally mapped their accumulations from 1987 to 2017.

### Core and edge areas

We calculated the Euclidean distance between the forest edge and its interior in GEE to assess changes in the amount of forest cores and edges for the PEC forests. We used a conservative threshold of 50 m to define forest edges since this is the distance in which the vegetation dynamics and structure are more affected by edge effects in the PEC [19, 48–50]. Using the core and edge layers, we calculated the total core and total edge area, as well as the proportions of forests covered by edges and core areas per year.

## Results

### Forest cover, number of fragments, average, and largest fragment sizes

**Fig 2:**
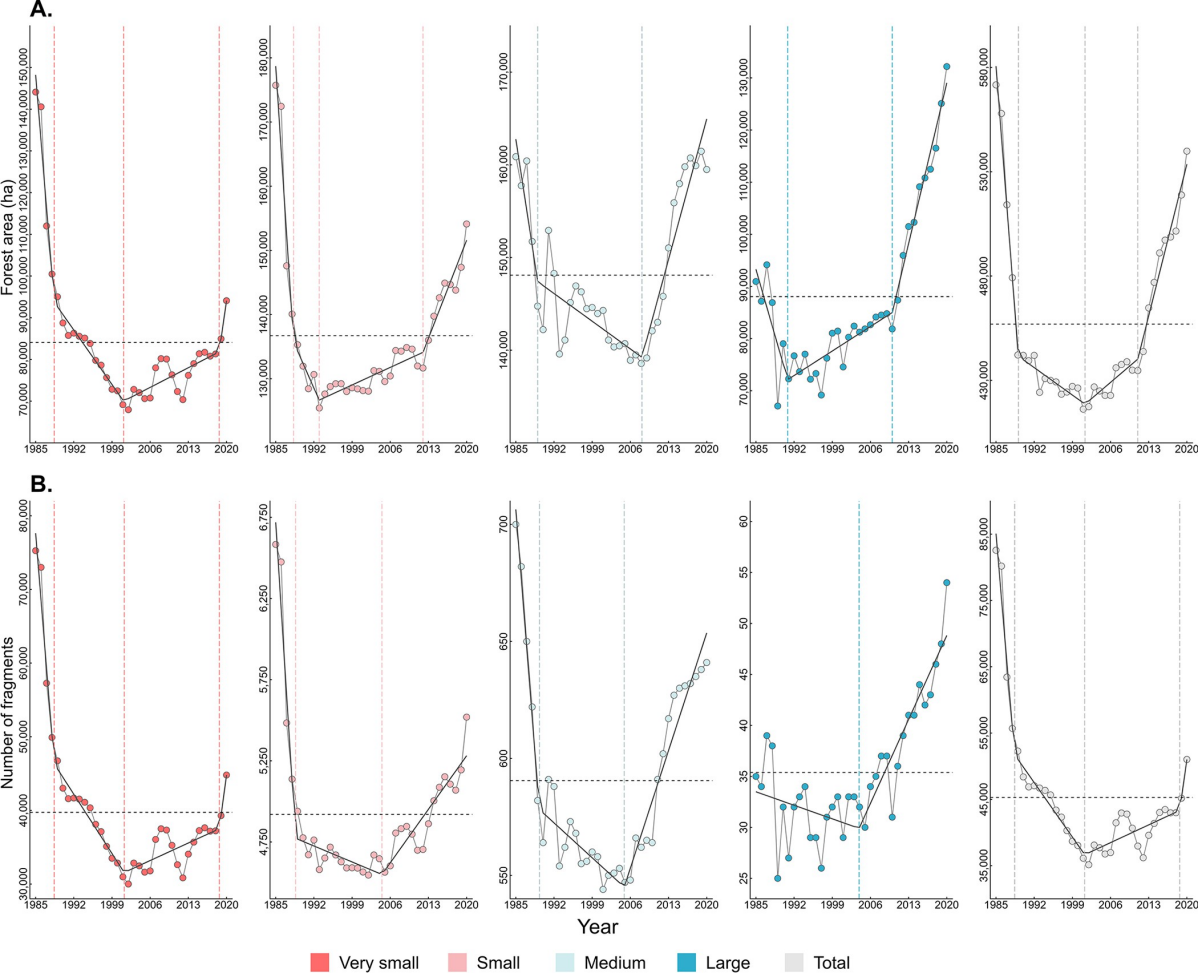
Historical changes in the forest cover of the Pernambuco Endemism Center. Section (A) displays changes in the total forest cover area (total and according to fragment size). Section (B) shows changes in the number of fragments (very small, small, medium, large, and total) from 1985 to 2020. Dotted horizontal lines represent the historical means and dotted vertical lines represent breakpoints. Bold black lines represent linear trends.

During the last decades, nearly 5% of the PEC forest cover was lost (571,661 ha in 1985 and 539,877 ha in 2020), and the most drastic reduction occurred before the 1990s (Fig 2A). The amount of forests we detected in 2020 represents 12.3% of the original PEC’s forest cover. Very small fragments (< 10 ha) represented around 25% (144,108 ha) of the forest cover in 1985 and 17% (94,091 ha) in 2020 (S1 Table). For forest areas, the first breakpoint was detected between 1988 and 1991 for total and all other classes of fragment size (Fig 2A), where higher rates of decrease were identified (S3 Table). Decreases in the forest extent were generally found to occur at a lower rate during the 1990s, followed by increases in the forest amount after the 2000s (S3 Table).

Almost all of the PEC fragments (roughly 87.44%) were classified as very small on average during the last decades, and only about 0.08% of the fragments were larger than 1,000 ha. Decreases in the number of fragments were found to be more pronounced during the 1980s, and the PEC experienced a period of lower decreasing rates during 1990 (S3 Table). After the 2000s, increases in the number of fragments were found for all fragment classes and total, with generally high rates of increase for medium and large fragments (S3 Table).

**Fig 3:**
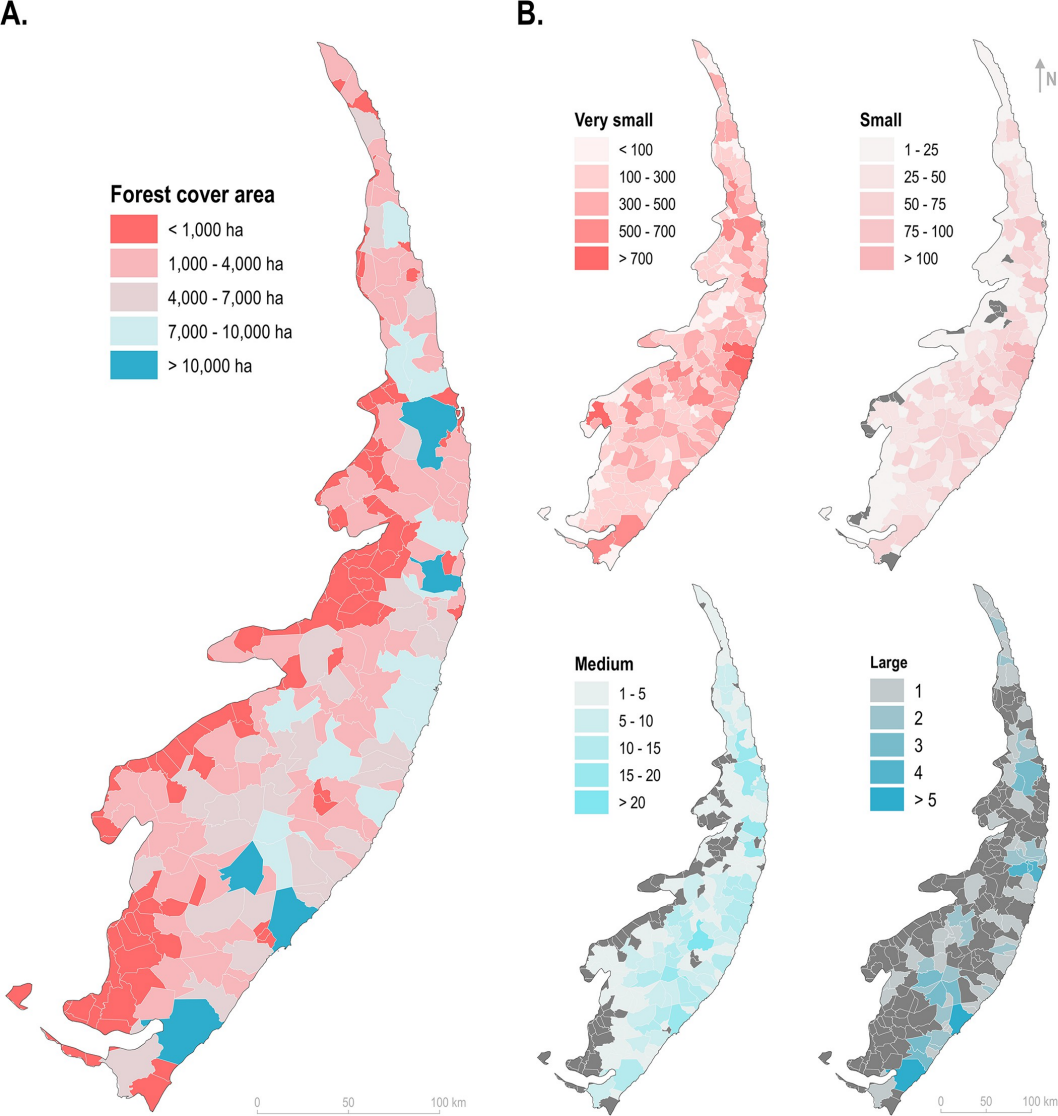
Current distribution of the forest cover and number of fragments of the Pernambuco Endemism Center. Current status of the (A) total forest cover and (B) the numbers of fragments according to their sizes: very small (< 10 ha), small (10–100 ha), medium (100–1,000 ha), and large (> 1,000 ha), for each municipality of the Pernambuco Endemism Center. Fragments located in borders between municipalities were accounted for each of them. The identification of the municipalities is provided in the Fig 1. The layer of administrative boundaries used in this map was obtained from the open-access dataset provided by the Brazilian Institute of Geography and Statistics (IBGE).

Only five municipalities of the PEC currently maintained more than 10,000 ha of native forests in 2020: Coruripe/AL, Maceió/AL, Murici/AL, Igarassu/PE, and Santa Rita/PB (Fig 3A). Fragments with increased sizes were generally confined to municipalities closer to the coast (eastern PEC) (Fig 3B). Furthermore, only the municipalities of Maceió and Coruripe in the state of Alagoas harbored more than five fragments larger than 1,000 ha each in 2020 (Fig 3B).

**Fig 4:**
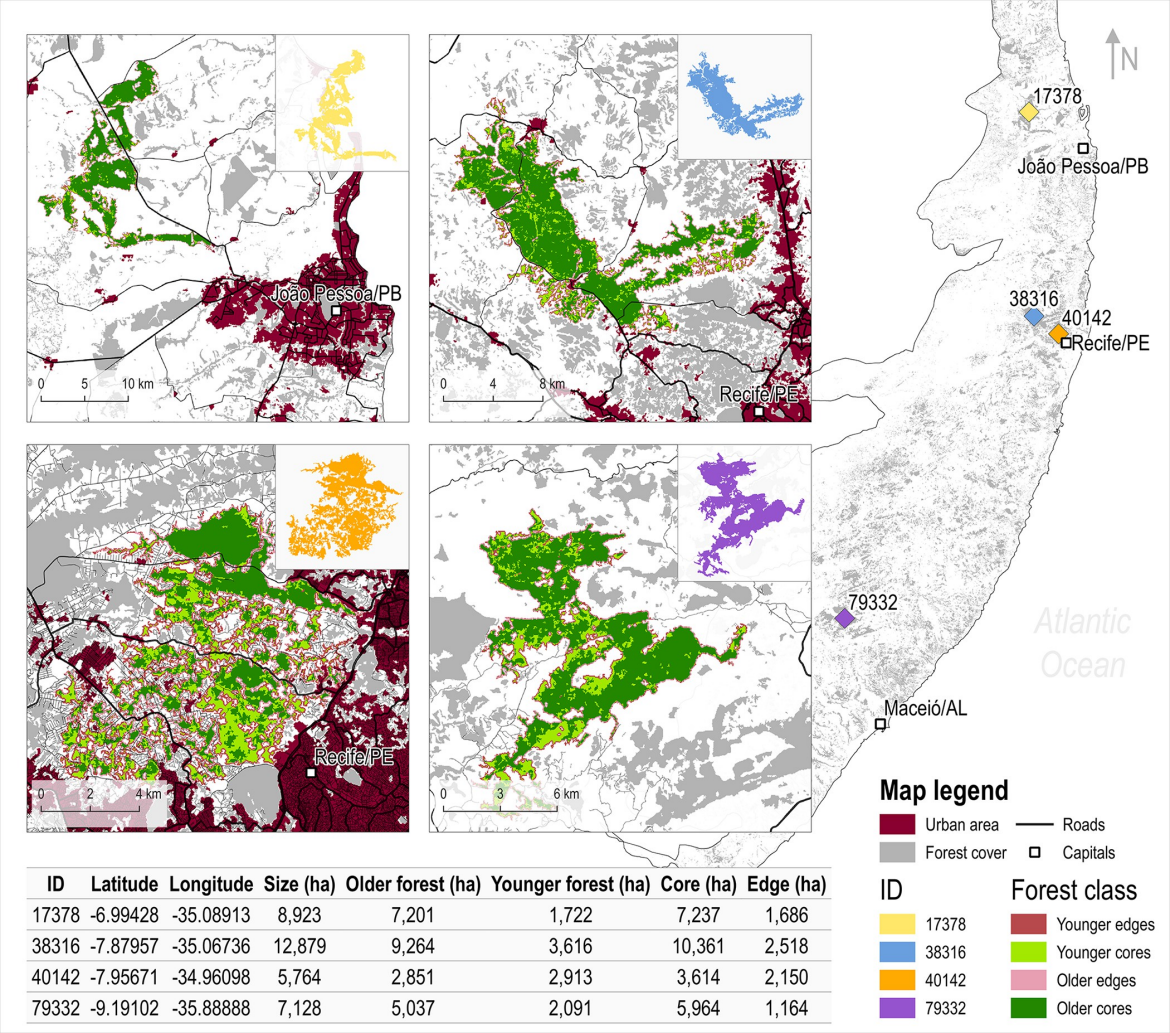
Largest forest fragments of the Pernambuco Endemism Center. Identification of the fragments larger than 5,000 ha in the year of 2017 over Pernambuco Endemism Center. The administrative boundaries, land cover, and federal road layers used in this map were obtained from the open-access datasets provided by the Brazilian Institute of Geography and Statistics (IBGE), MapBiomas Project, and Brazilian Water Agency (ANA), respectively.

The average size of the PEC’s fragments was roughly 10.37 ha over the last decades and a significant increase was observed over time (0.11 ha/yr^-1^, R^2^ = 0.68; p-value < 0.001). The average size of the PEC’s largest fragment over time was around 11,812 ha, and in 2020 the largest fragment was bigger than 14,000 ha for the first time since 1986. We identified four forest fragments larger than 5,000 ha in 2017, located in the states of Alagoas (ID 79332), Pernambuco (IDs 38316, 40142), and Paraíba (ID 17378) (Fig 4). The largest fragment identified in 2017 was ID 388316, located in Pernambuco state, with 12,897 ha (Fig 4). All fragments except ID 40142 were primarily composed by older forests that grew before 1985 (ID 79332: 71%; ID 40142: 49%; ID 38316: 72%; ID 17378: 81%) (Fig 4). In addition, ID 40142 also presented the lowest percentage of core area cover (63%). However, except for ID 79332, all fragments larger than 5,000 ha were located nearby roads and state capitals (Fig 4).

### Identification of older and younger forests

We identified three municipalities in Alagoas state (Coruripe, Maceió, and Murici), three in Pernambuco (Água Preta, Abreu e Lima, and Igarassu), and two in Paraíba (Santa Rita and Rio Tinto) with more than 5,000 ha of older forests. In summary, we detected a current cover of 237,708 ha of older forests and 260,984 ha of younger forests in 2017 (S5 Fig).

### Deforestation and forest regeneration

**Fig 5:**
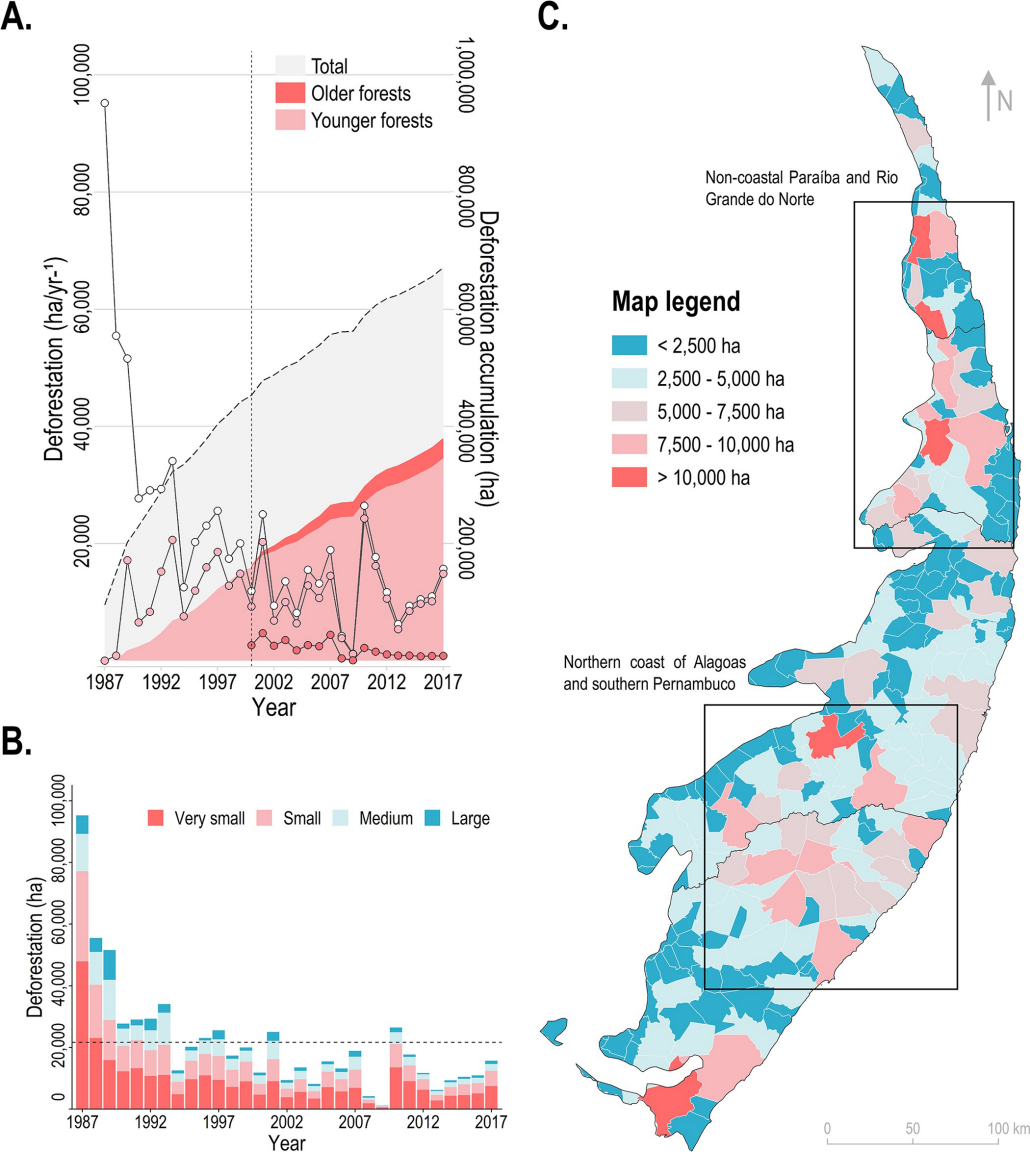
Forest losses over the last decades in the Pernambuco Endemism Center. Section (A) shows an overview of the annual deforestation rates (total, older, and younger forest losses) and the deforestation accumulation from 1987 to 2017. Section (B) displays the losses in very small (< 10 ha), small (10–100 ha), medium (100–1,000 ha), and large (> 1,000 ha) fragments. The dotted horizontal line is the average annual deforestation rate. Section (C) shows an overview of the deforestation accumulation for each municipality of the PEC, from 1987 to 2017. The municipalities’ identification is provided in Fig 1. The layer of administrative boundaries used in this map was obtained from the open-access dataset provided by the Brazilian Institute of Geography and Statistics (IBGE).

We identified the accumulated loss of more than 670,000 ha of forests in the PEC over the last decades (Fig 5A). The average annual deforestation rate was around 21,648 ha/yr^-1^, and the highest losses were observed before the 1990s (1987: 95,198 ha; 1988: 55,486 ha; 1989: 51,595 ha) (Fig 5A). After the 2000s, deforestation rates were mainly below the average annual deforestation rate (Fig 5A). The deforestation rate of older forests was around 1,903 ha/yr^-1^ since 2000 and of younger forests was roughly 11,159 ha/yr^-1^ since 1987 (Fig 5A). Deforestation was more common in very small fragments; however, we detected evidence of losses of 20,138 ha in large fragments from 1987 to 1989 (Fig 5B).

Five municipalities (Penedo/AL, Bonito/PE, Sapé/PB, Pedro Velho/RN, and São José de Mipibu/RN) lost over 10,000 ha of forests since the late 1980s (Fig 5C). Deforestation was largely concentrated in the municipalities near the borders between northern Alagoas and southern Pernambuco, and in a latitudinal gradient extending from the non-coastal PEC regions of Paraíba to middle Rio Grande do Norte (Fig 5C).

**Fig 6:**
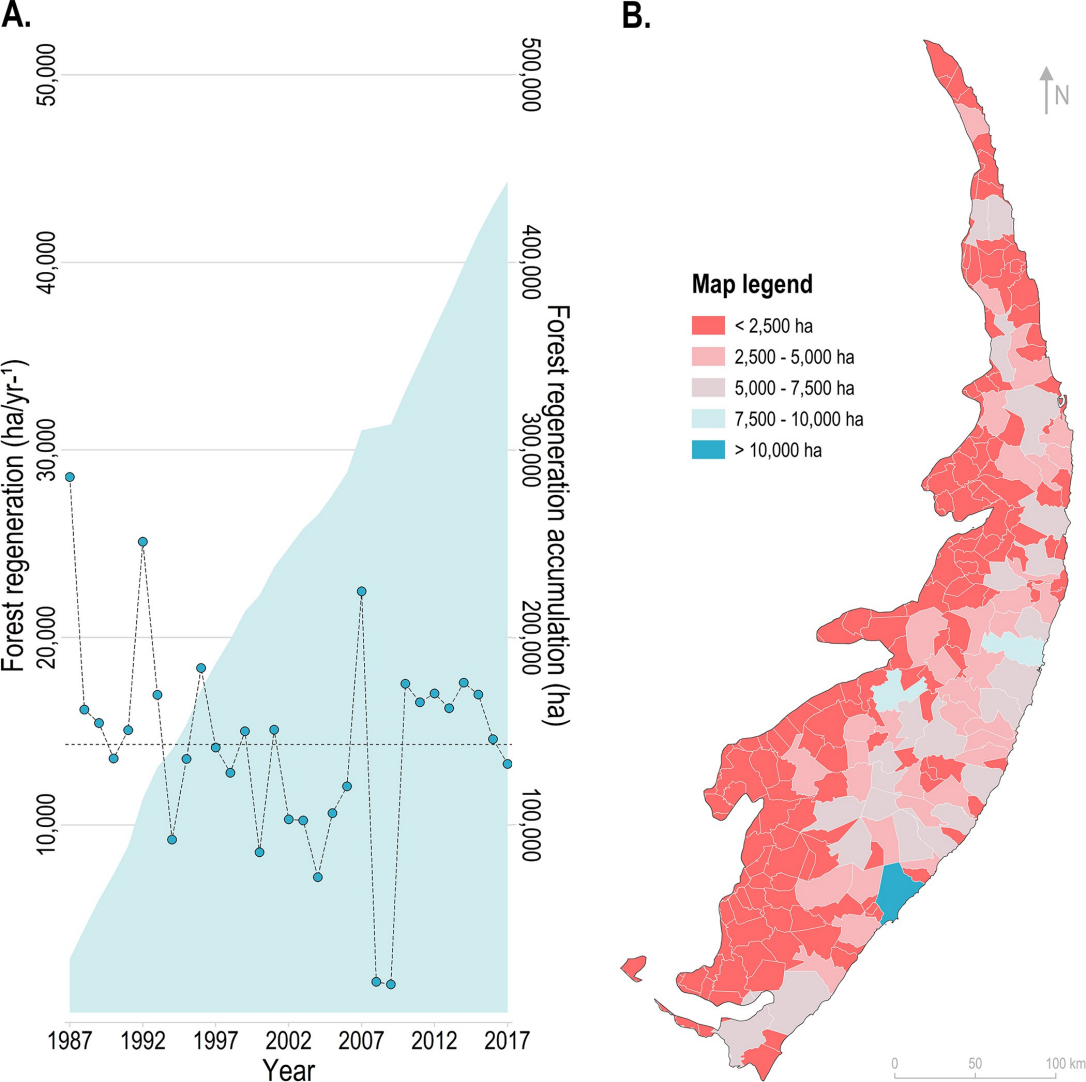
Forest regeneration over the last decades in the Pernambuco Endemism Center. Section (A) displays annual forest regeneration rates and the accumulation of forest gain over time. The dotted horizontal line represents the average annual forest regeneration rate. Section (B) shows the spatial distribution of forest regeneration accumulation across the municipalities of the PEC. The municipalities’ identification is provided in Fig 1. The layer of administrative boundaries used in this map was obtained from the open-access dataset provided by the Brazilian Institute of Geography and Statistics (IBGE).

Over the last few decades, the PEC experienced the accumulation of 443,324 ha of forest regeneration, with an average annual rate of 14,301 ha/yr^-1^ (Fig 6A). In general, higher reforestation rates were detected during the first years of our time-lapse (Fig 6A). The municipalities of Maceió/AL, Bonito/PE, and Cabo de Santo Agostinho/PE accumulated the highest forest regeneration rates since 1987 (Fig 6B).

### Core and edge areas

**Fig 7:**
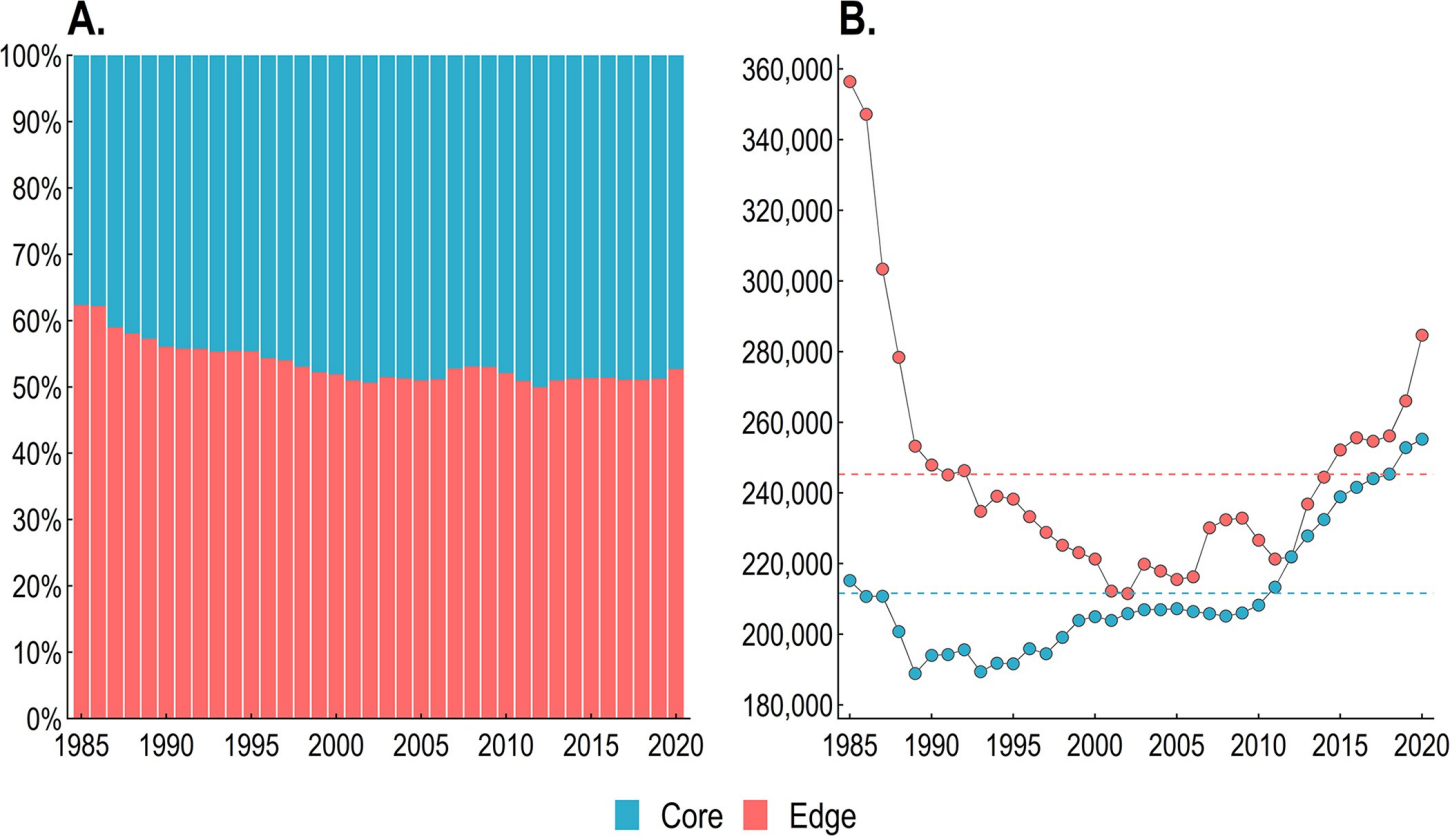
Historical changes in core and edge areas of the Pernambuco Endemism Center. Section (A) displays the variation in the percentage of forests composed of cores and edges over time. Section (B) shows changes in the area (ha) of cores and edges. The bluish and reddish horizontal dotted lines in Section B represent the average annual areas of cores and edges, respectively.

On average, more than half of the PEC forest cover (53.6% ± 3.2%) was located within the first 50 m from the edge, with mean core and edge areas being around 211,615 ha and 245,314 ha, respectively. We found about 10% of increase in the proportion of core areas over our study period. Currently, the total forest cover represented by core and edge areas are 255,228 ha and 284,649 ha, respectively (Fig 7).

### Forest quality

The amount of older forest cores in large fragments, which may represent the PEC’s higher-quality habitats, was only about 62,058 ha in 2017, representing 12% of the total forest cover in that year (Table 1 and S6 Fig).

**Table 1:** Area of the Atlantic Forest cover over the Pernambuco Endemism Center according to quality.

	Very small	Small	Medium	Large
**Older forest cores**	2,501 ha	33,692 ha	67,848 ha	62,058 ha
**Younger forest cores**	2,902 ha	21,79 ha	29,737 ha	18,775 ha
**Older forest edges**	11,319 ha	25,729 ha	21,284 ha	11,137 ha
**Younger forest edges**	47,805 ha	51,369 ha	34,601 ha	16,438 ha

Forest Area of older and younger forests distributed into cores and edges in very small (< 10 ha), small (10–100 ha), medium (100–1,000 ha), and large (> 1,000 ha) fragments of the Pernambuco Endemism Center in 2017.

## Discussion

Our main finding is that a large portion of the PEC forests is threatened not only by fragmentation and edge effects but also by forest rejuvenation, with older forest cores in large fragments representing only 12% (62,058 ha) of the remaining forest cover in 2017. Temporal analyses revealed an overall reduction of around 5% in the total forest cover from 1985 to 2020, and the highest deforestation rates were found between 1985 and the early 2000s. At least 87% of the 4.4 Mha of the original PEC forest cover was devastated before 1985, suggesting that most of its fragments may have been isolated for many decades or centuries. Although we observed a tendency for forest cover recuperation in the last two decades, it was insufficient to compensate for the losses that occurred in the decades of 1980 and 1990. Our estimate of the total remaining forest cover of the PEC was higher than previously reported, i.e., in 1990–1995 (256,581 ha) [30], 2005 (360,455 ha) [22], and 2001–2007 (322,372 ha) [29]. We suggest that it has occurred due to: i) the recuperation of part of the forests during the last decade, ii) differences in the data spatial resolution, iii) differences in the minimum size of fragments considered as forests, and iv) the use of more conservative methods for forest cover classification in the abovementioned studies. The situation observed in the PEC may also extend to other tropical forests in human-modified landscapes, and our results suggest the need for further investigations to characterize the degradation of these megadiverse ecosystems around the globe.

We observed a positive balance between forest regeneration and deforestation and a decrease in forest fragmentation in the last decade. This tendency may be related to the PEC’s ongoing initiatives of forest restoration, linked to the Atlantic Forest Restoration Pact [23] and/or changes in practices of the sugar-cane industry in the face of the increasing need for producing “environmentally correct” products since the 1990s [24]. However, this should not obscure the dramatic conservation status of the PEC forests. As expected, the drastic deforestation of the decades of 1980 and 1990, and the tendency of forest regeneration registered during the last decade, resulted in a process of forest rejuvenation about 2.5 times higher than that estimated for the whole Brazilian Atlantic Forest [16]. Despite the importance of secondary forests in maintaining a fraction of the original biological diversity in certain regions [51–53], younger forests may not maintain high-quality habitats, and their ecological communities can be altered [54–56]. In the PEC, areas that have been through restoration programs, for instance, were only a third as dense as older forest remnants and maintained considerably different tree communities, with only half of the original vegetal species richness [57].

It is also of great concern that the PEC forests are currently mainly distributed (roughly 90%) into very small fragments (< 10 ha) and that the average fragment size is only about 11 ha. This scenario is more pessimistic than previous reports that informed that 73.3% of the PEC fragments were smaller than 10 ha [29]. Our findings were also more alarming than the previous estimates for the entire Brazilian Atlantic Forest, in which 80% of fragments were smaller than 50 ha [22]. The mean fragment size of the PEC was also smaller than the overall values observed for tropical forests (17 ha for the Americas and 13 ha for Asia and Australia) [58].

Nowadays, only about 12% (62,058 ha) of the PEC forest cover is composed of higher-quality habitats (i.e., older forest cores in fragments larger than 1,000 ha). It is worth noting, however, that we considered as older, the forests present in 1985 not removed until 2017, meaning that we have not discriminated between the areas targeted to selective logging and those that could have regenerated just before 1985. Then, the amount of primary areas is certainly smaller than our estimate. This may be the reason why old-growth forests with full capacity of carbon storage represent only 8% of the total forest cover of the PEC [59, 60]. Furthermore, we only evaluated edges effects within the first 50 m from the borders. However, forest-dwelling and niche specialized tropical species tend to be highly sensitive to edge effects, which can extend up to 400 m into the interior of tropical forests [61–66].

The scenario of intense forest rejuvenation and fragmentation highlights the importance of maintaining the last older core areas in larger fragments of the PEC to preserve taxa dependent on older forests to thrive and to serve as sources of biodiversity for the regenerating areas. We suggest that ensuring effective protection of these larger blocks of older forest cores and increasing connectivity to their surrounding forests must be top priorities for the conservation of the PEC forest-dependent biodiversity. The increase in metrics related to habitat quality over the last decades may indicate a slight recovery of the native forest cover in the region, but forest rejuvenation, fragmentation, and edge effects are still undergoing threats to the PEC. Our results evidenced that forest cover information alone may provide a false scenario about the conservation status of the Brazilian Atlantic Forest. By addressing the temporal component and investigating the spatial characteristics of the fragments, we provide a more realistic scenario of the PEC’s forest cover and more precise information for conservation practitioners and decision-makers.

## Supporting information

S1 Fig Moving window temporal filter for classifying deforestation. (TIF)

S2 Fig Moving window temporal filter for classifying forest regeneration. (TIF)

S3 Fig Scheme of the methods for the classification of deforestation according to fragment size. (TIF)

S4 Fig Scheme for the classification of older forests deforestation. (TIF)

S5 Fig Older and younger forests of the Pernambuco Endemism Center. The current distribution of older and younger forests according to (A) the municipalities of the PEC, and (B) the spatial distribution and configuration of older and younger forests. The layer of administrative boundaries used in this map was obtained from the open-access dataset provided by the Brazilian Institute of Geography and Statistics (IBGE).
(TIF)

S6 Fig Quality of the Atlantic Forest cover over the Pernambuco Endemism Center. Current (2017) classification of forests according to age and edge effects for (A) large (> 1,000 ha), and (B) medium (100–1,000 ha) fragments over the Pernambuco Endemism Center.
(TIF)

S1 File (DOCX)

S1 Table Landscape metrics of forests over the Pernambuco Endemism Center. Class-level metrics related to forest cover area (total and by four classes of fragment size), number of fragments (total and by four classes of fragment size), largest fragment area, mean fragment area, core and edge areas. Values presented in hectares for area-related metrics.
(DOCX)

S2 Table Deforestation and forest regeneration over the Pernambuco Endemism Center. Deforestation was classified according to fragment size (deforestation of very small, small, medium, and large fragments) and forest age (in deforestation of older and younger forests). Values presented in hectares.
(DOCX)

S3 Table Breakpoints and linear trends of forest cover area and number of fragments of the Pernambuco Endemism Center. Forests were classified according to fragment size (very small, small, medium, and large fragments), and metrics were calculated separately for all of them and total. Values for areas presented in hectares.
(DOCX)
