# Supplementary figures and images for: Spatiotemporal dynamics reveals forest rejuvenation, fragmentation, and edge effects in an Atlantic Forest hotspot, the Pernambuco Endemism Center, northeastern Brazil
Source: PLoS One. 2023 Sep 8;18(9):e0291234. doi: 10.1371/journal.pone.0291234 (PMC10490850; doi:10.1371/journal.pone.0291234)

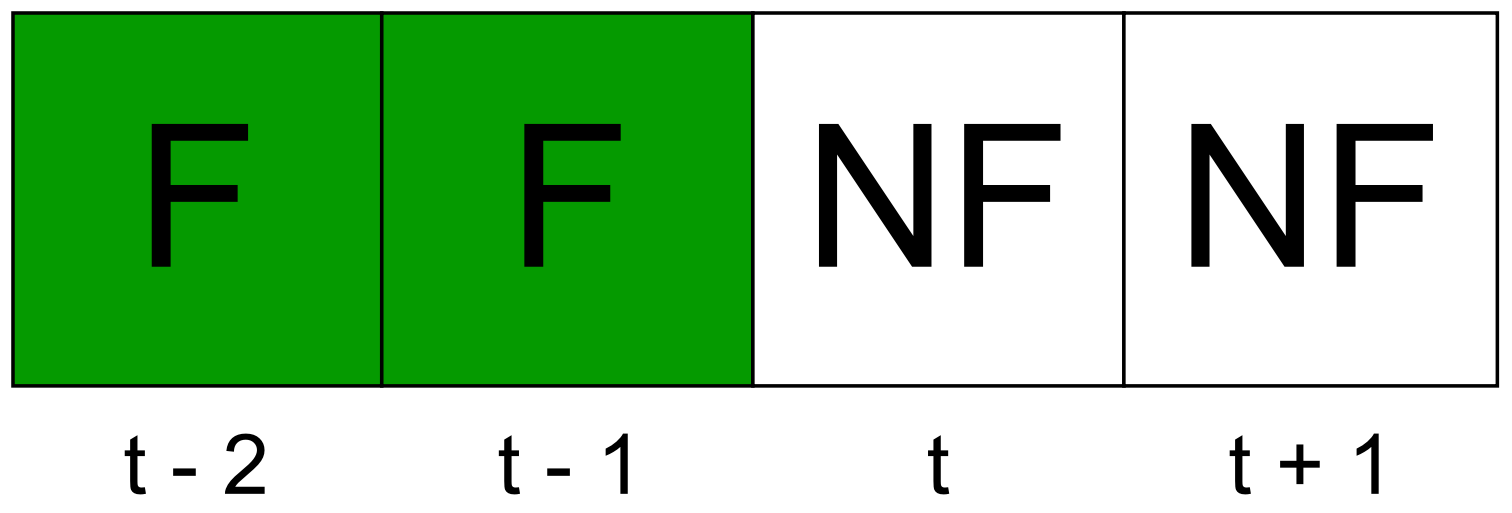

Supplement: S1 Fig — (TIF) [file pone.0291234.s001.tif]

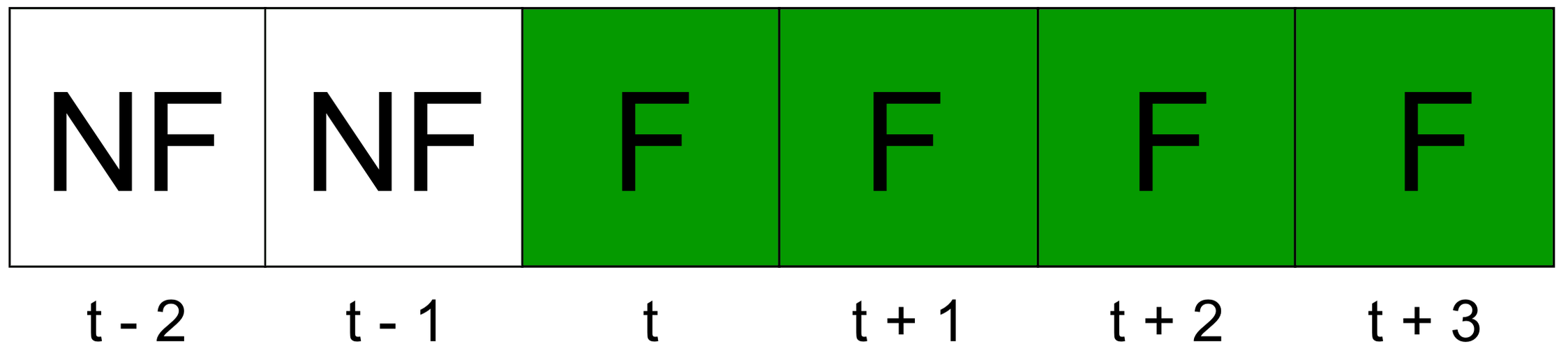

Supplement: S2 Fig — (TIF) [file pone.0291234.s002.tif]

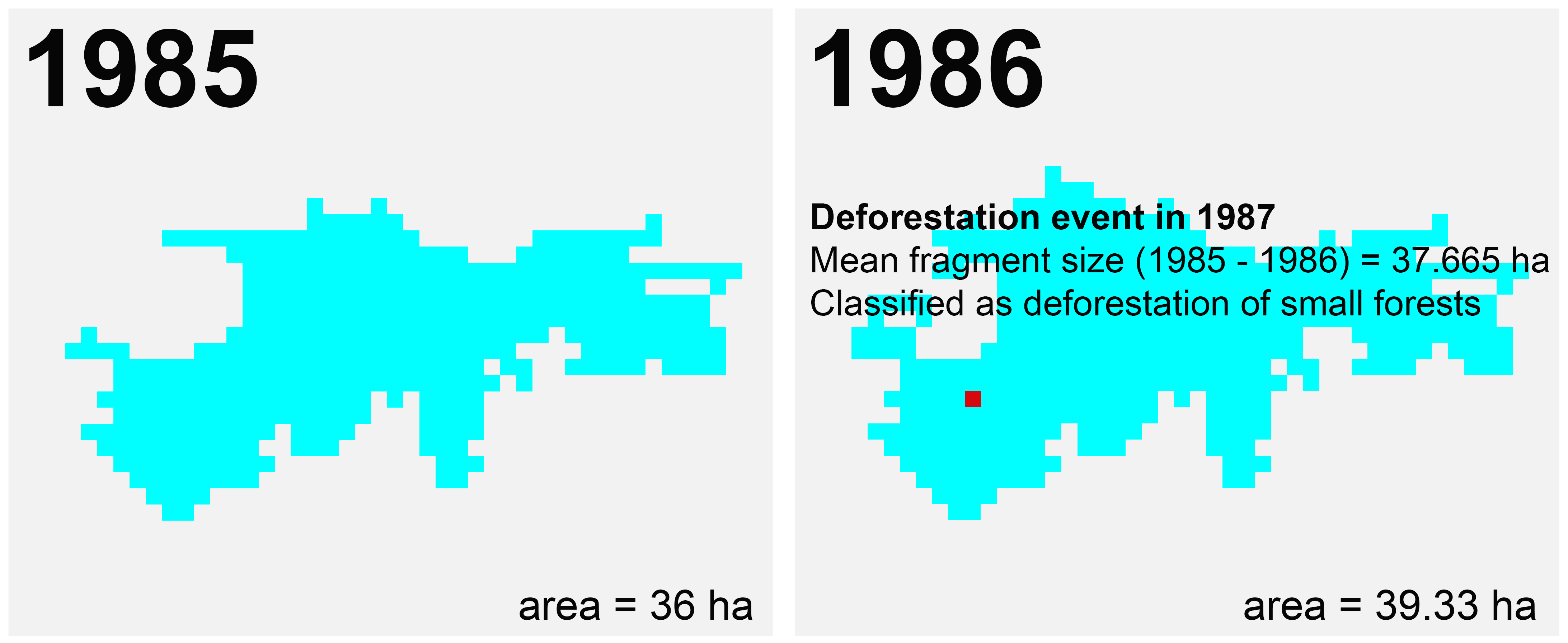

Supplement: S3 Fig — (TIF) [file pone.0291234.s003.tif]

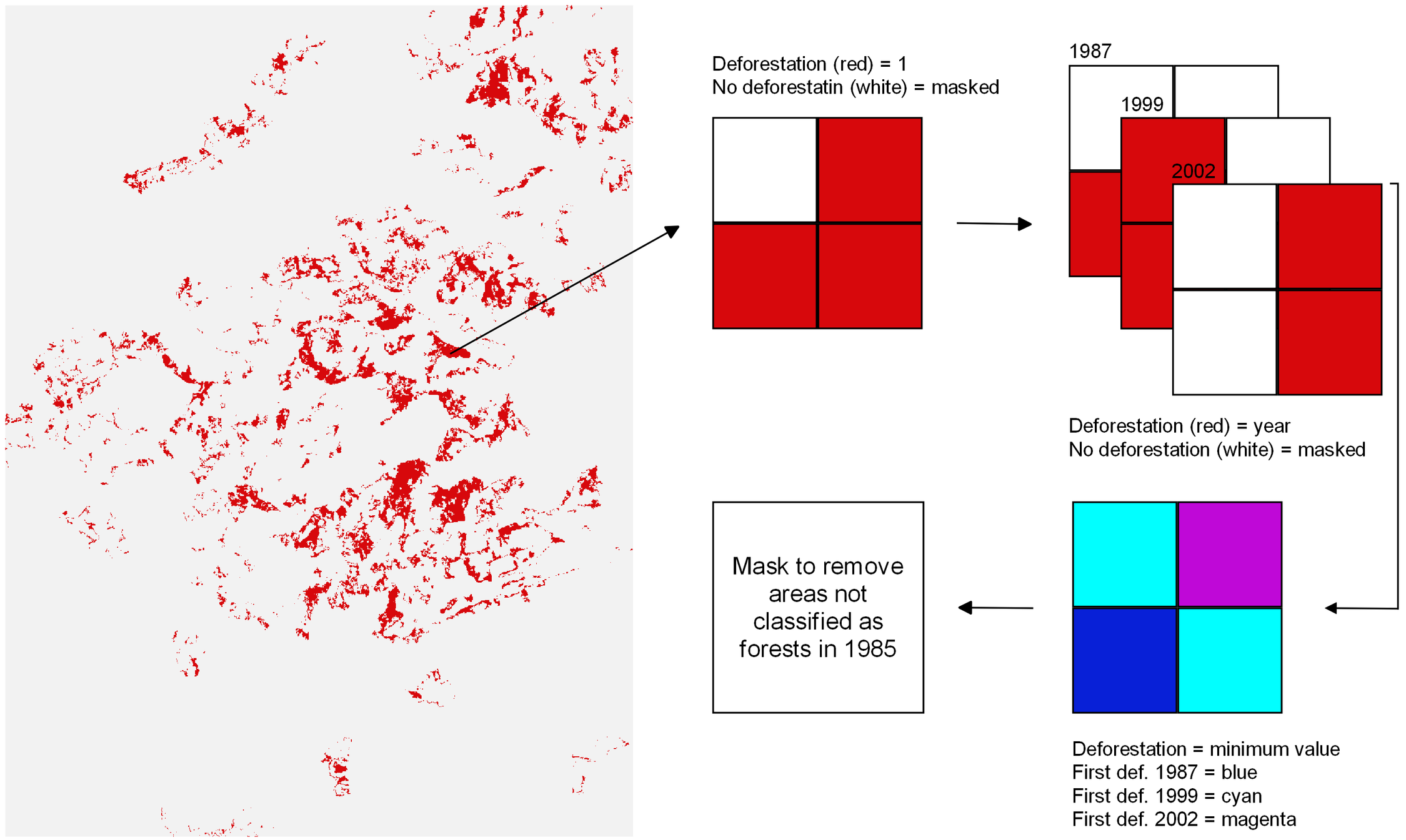

Supplement: S4 Fig — (TIF) [file pone.0291234.s004.tif]

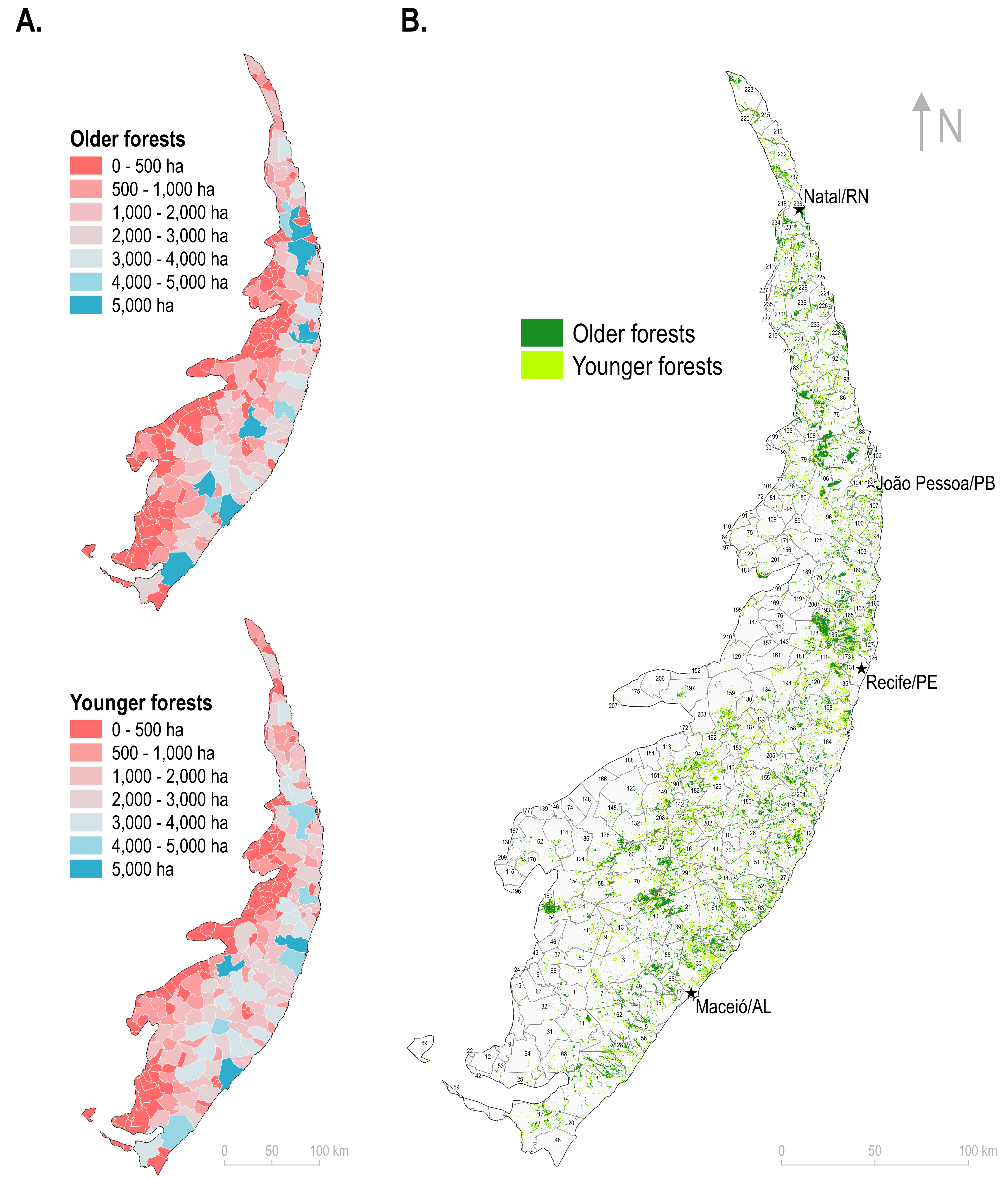

Supplement: S5 Fig — The current distribution of older and younger forests according to (A) the municipalities of the PEC, and (B) the spatial distribution and configuration of older and younger forests. The layer of administrative boundaries used in this map was obtained from the open-access dataset provided by the Brazilian Institute of Geography and Statistics (IBGE). (TIF) [file pone.0291234.s005.tif]

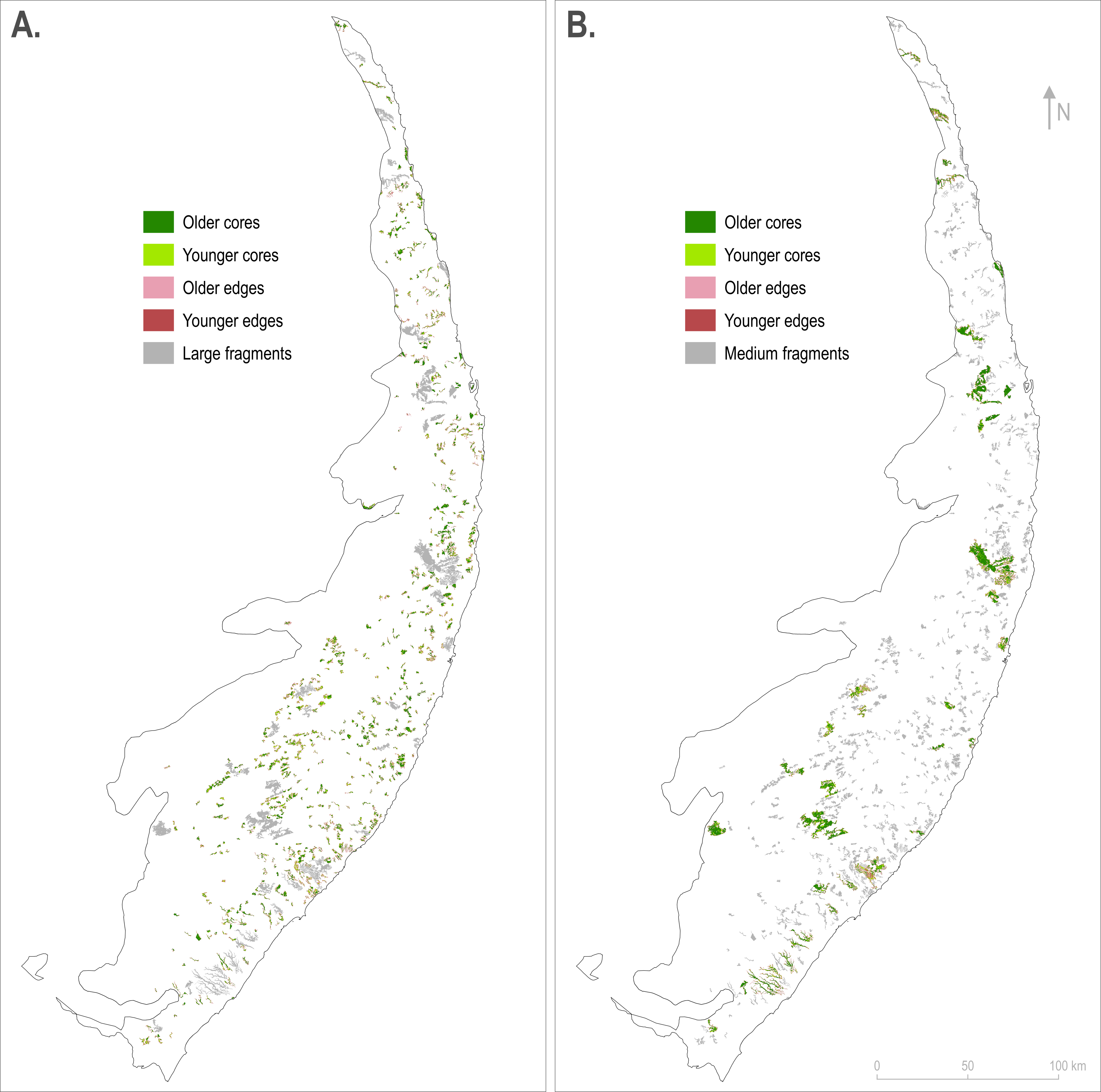

Supplement: S6 Fig — Current (2017) classification of forests according to age and edge effects for (A) large (> 1,000 ha), and (B) medium (100–1,000 ha) fragments over the Pernambuco Endemism Center. (TIF) [file pone.0291234.s006.tif]
