# Supplementary material for: Spatiotemporal dynamics reveals forest rejuvenation, fragmentation, and edge effects in an Atlantic Forest hotspot, the Pernambuco Endemism Center, northeastern Brazil
Source: PLoS One. 2023 Sep 8;18(9):e0291234. doi: 10.1371/journal.pone.0291234 (PMC10490850; doi:10.1371/journal.pone.0291234)
Supplement: S1 File — (DOCX) [file pone.0291234.s007.docx]

# Part 1. Assessing, filtering, and export MapBiomas data to Google Drive using Google Earth Engine

## // First, upload a shapefile of your region of interest and name the object as “ROI”

## // Loading data from MapBiomas Collection 6.0, clip it to your region of interest, and keep only class 3 (“forest formation”)

var landCover = ee.Image('projects/mapbiomas-workspace/public/collection6/mapbiomas_collection60_integration_v1')

.clip(ROI).eq(3);

## // Since MapBiomas data comes in ee.Image format (where each band represents data from annual land cover classifications), convert it to ee.ImageCollection

landCover = ee.ImageCollection([landCover.select('classification_1985'), landCover.select('classification_1986'),

landCover.select('classification_1987'), landCover.select('classification_1988'),

landCover.select('classification_1989'), landCover.select('classification_1990'),

landCover.select('classification_1991'), landCover.select('classification_1992'),

landCover.select('classification_1993'), landCover.select('classification_1994'),

landCover.select('classification_1995'), landCover.select('classification_1996'),

landCover.select('classification_1997'), landCover.select('classification_1998'),

landCover.select('classification_1999'), landCover.select('classification_2000'),

landCover.select('classification_2001'), landCover.select('classification_2002'),

landCover.select('classification_2003'), landCover.select('classification_2004'),

landCover.select('classification_2005'), landCover.select('classification_2006'),

landCover.select('classification_2007'), landCover.select('classification_2008'),

landCover.select('classification_2009'), landCover.select('classification_2010'),

landCover.select('classification_2011'), landCover.select('classification_2012'),

landCover.select('classification_2013'), landCover.select('classification_2014'),

landCover.select('classification_2015'), landCover.select('classification_2016'),

landCover.select('classification_2017'), landCover.select('classification_2018'),

landCover.select('classification_2019'), landCover.select('classification_2020')]);

## // Map it through the ee.ImageCollection to assign non-values to areas not classified as forests

landCover = landCover.map(function(img){

return(img.updateMask(img.eq(1)));

});

## // You may want to check your collection by printing it and adding the first image to the map

//print(landCover)

//Map.addLayer(landCover.first())

## // Batch download all images

var batch = require('users/fitoprincipe/geetools:batch');

batch.Download.ImageCollection.toDrive(landCover, 'GEE', {

name: 'mapbiomas_{id}',

scale: 30,

region: roi,

maxPixels: 1e13,

crs: 'EPSG:4326',

fileFormat: "GeoTIFF",

type: 'float'

});

# Part 2. Assigning individual numbers and calculating size of forest patches using R

## ##### Function from the original clumpSize function in bFastSpatial package by Ben DeVries #####

## ##### GitHub: <https://github.com/loicdtx/bfastSpatial> #####

clumpSize <- function(x, f=1, stats=FALSE, ...){

# Identify the clumps

y <- clump(x, directions=4, ...)

# Make a reclassification matrix from the frequency table of y

rcl <- freq(y)

# Conversion to other unit if factor is supplied

if(f != 1)

rcl[, 2] <- rcl[, 2] * f

# Optional: make a summary matrix

if(stats){

sumstat <- matrix(nc=1, nr=6, dimnames=list(c("Mean", "Min.", "1st Qu.", "Median", "3rd Qu.", "Max."), c("clump size")))

sizes <- rcl[!is.na(rcl[, 1]), 2]

sumstat[1, 1] <- mean(sizes)

sumstat[c(2:6), 1] <- quantile(sizes)

}

# Reclassify y based on rcl

rcl <- cbind(rcl[,1], rcl) # double first column (see ?reclassify)

z <- reclassify(y, rcl=rcl, right=NA)

# Remove NAs

z[is.na(y)] <- NA

# make z into a list if stats=TRUE

if(stats)

z <- list(clumps = z, stats = sumstat)

return(stack(y, z))

}

## ##### Loading packages and setting preferences #####

library(raster)

rasterOptions(maxmemory=1e+06, chunksize=1e+07, progress = 'text'); message("Memory preferences set.")

## ##### Loading directories of the folder with the forest cover classification images #####

(files <- list.files(path="pathtoyourdata", pattern=".tif", full.names=TRUE))

## ##### Stacking #####

forestCover <- stack(files)

## ##### Applying the function #####

for(i in 1:nlayers(forestCover)){

forestLayer <- clumpSize(forestCover[[i]])

writeRaster(forestLayer, paste0('pathtosave', 'clump_', names(forestCover[[i]]), '.tif'), format="GTiff")

rm(forestLayer)

#gc() # use it if you are running out of memory

#Sys.sleep(10) # use it and adjust the time in seconds if you are running out of memory

}

# Part 3. Calculating metrics and investigating the spatial distribution of forests using Google Earth Engine

## /*

## First, upload the images generated in the previous R script to GEE, load them into your workspace, and name them

## as "image1", "image2", ..., "image36". The first band of the images (named 'b1') should be representative of the

## unique IDs assigned to each clump in R. The second band (named 'b2') should represent the clump size in number

## of pixels. Then, load your region of interest and name it as "ROI". Lastly, load the municipalities shapefile

## (or another shapefile representative of the scale you want to capture the processes related to forest dynamics) and

## name it as "features".

## */

## // Adding data as one of the properties of your images

image1 = image1.set('system:time_start', ee.Date('1985')); image2 = image2.set('system:time_start', ee.Date('1986'));

image3 = image3.set('system:time_start', ee.Date('1987')); image4 = image4.set('system:time_start', ee.Date('1988'));

image5 = image5.set('system:time_start', ee.Date('1989')); image6 = image6.set('system:time_start', ee.Date('1990'));

image7 = image7.set('system:time_start', ee.Date('1991')); image8 = image8.set('system:time_start', ee.Date('1992'));

image9 = image9.set('system:time_start', ee.Date('1993')); image10 = image10.set('system:time_start', ee.Date('1994'));

image11 = image11.set('system:time_start', ee.Date('1995')); image12 = image12.set('system:time_start', ee.Date('1996'));

image13 = image13.set('system:time_start', ee.Date('1997')); image14 = image14.set('system:time_start', ee.Date('1998'));

image15 = image15.set('system:time_start', ee.Date('1999')); image16 = image16.set('system:time_start', ee.Date('2000'));

image17 = image17.set('system:time_start', ee.Date('2001')); image18 = image18.set('system:time_start', ee.Date('2002'));

image19 = image19.set('system:time_start', ee.Date('2003')); image20 = image20.set('system:time_start', ee.Date('2004'));

image21 = image21.set('system:time_start', ee.Date('2005')); image22 = image22.set('system:time_start', ee.Date('2006'));

image23 = image23.set('system:time_start', ee.Date('2007')); image24 = image24.set('system:time_start', ee.Date('2008'));

image25 = image25.set('system:time_start', ee.Date('2009')); image26 = image26.set('system:time_start', ee.Date('2010'));

image27 = image27.set('system:time_start', ee.Date('2011')); image28 = image28.set('system:time_start', ee.Date('2012'));

image29 = image29.set('system:time_start', ee.Date('2013')); image30 = image30.set('system:time_start', ee.Date('2014'));

image31 = image31.set('system:time_start', ee.Date('2015')); image32 = image32.set('system:time_start', ee.Date('2016'));

image33 = image33.set('system:time_start', ee.Date('2017')); image34 = image34.set('system:time_start', ee.Date('2018'));

image35 = image35.set('system:time_start', ee.Date('2019')); image36 = image36.set('system:time_start', ee.Date('2020'));

## // Removing patches with less than 6 pixels (0.54 ha)

var myCollection = ee.ImageCollection([

image1,image2,image3,image4,image5,image6,image7,image8,image9,image10,image11,image12,image13,

image14,image15,image16,image17,image18,image19,image20,image21,image22,image23,image24,image25,

image26,image27,image28,image29,image30,image31,image32,image33,image34,image35,image36]).map(

function(image){

return image.updateMask(image.select('b2').gte(6));

}

);

## // Adding a band with forest cover = 1 and a band of area in hectare, multiply number of pixels by pixel size in ha (0.09)

myCollection = myCollection.map(function(image){

return image.addBands(image.select('b2').where(image.select('b2'), 1))

.addBands(image.select('b2').multiply(0.09))

.clip(ROI)

.rename(['id', 'nPixels', 'fCover', 'pSize_ha']);

});

## // Use this if you need to fix geometries of the "features" object

/*

features = features.map(function (feature) {

var filteredGeoms = feature.geometry().geometries().map(function (geometry) {

geometry = ee.Geometry(geometry);

return ee.Algorithms.If(geometry.type().compareTo('Polygon'), null, geometry);

}, true);

return feature.setGeometry(ee.Geometry.MultiPolygon(filteredGeoms));

});

*/

## /*********************************************************************************************/

## /********************************** Class-level Landscape Metrics *********************************/

## /*********************************************************************************************/

### // Generating bands for calculaling class-level landscape metrics through GEE reducers

var patchMetrics = myCollection.map(function(image){

### // Area of very small (< 10 ha) fragments

var vs_ha = image.select('pSize_ha').updateMask(image.select('pSize_ha').lt(10));

vs_ha = vs_ha.where(vs_ha, 1);

### // Area of small (10 - 100 ha) fragments

var s_ha = image.select('pSize_ha').updateMask(image.select('pSize_ha').gte(10).and(image.select('pSize_ha').lt(100)));

s_ha = s_ha.where(s_ha, 1);

### // Area of medium (100 - 1,000 ha) fragments

var m_ha = image.select('pSize_ha').updateMask(image.select('pSize_ha').gte(100).and(image.select('pSize_ha').lt(1000)));

m_ha = m_ha.where(m_ha, 1);

### // Area of large (> 1,000 ha) fragments

var l_ha = image.select('pSize_ha').updateMask(image.select('pSize_ha').gte(1000));

l_ha = l_ha.where(l_ha, 1);

### // Number of very small (< 10 ha) fragments

var vs_id = image.select('id').updateMask(image.select('pSize_ha').lt(10));

### // Number of small (10 - 100 ha) fragments

var s_id = image.select('id').updateMask(image.select('pSize_ha').gte(10).and(image.select('pSize_ha').lt(100)));

### // Number of medium (100 - 1,000 ha) fragments

var m_id = image.select('id').updateMask(image.select('pSize_ha').gte(100).and(image.select('pSize_ha').lt(1000)));

### // Number of large (> 1,000 ha) fragments

var l_id = image.select('id').updateMask(image.select('pSize_ha').gte(1000));

### // Area of cores and edges

var mask = image.select('fCover').unmask().clip(ROI);

var forest = mask.eq(1);

var nonForest = mask.neq(1);

var dist = forest.cumulativeCost({

source: nonForest.updateMask(nonForest),

maxDistance: 50000

}).updateMask(image

.select('fCover')

.eq(1))

.rename('distToEdge')

.reproject({

crs: image.select('id').projection(), scale: 30

}).reduceResolution({

reducer: ee.Reducer.max()

});

var core = dist.updateMask(dist.gt(50)).where(dist.updateMask(dist.gt(50)), 1);

var edge = dist.updateMask(dist.lte(50)).where(dist.updateMask(dist.lte(50)), 1);

core = core.updateMask(image.select('fCover'));

edge = edge.updateMask(image.select('fCover'));

// Adding the new bands to the images

return image.addBands(vs_ha).addBands(s_ha).addBands(m_ha)

.addBands(l_ha).addBands(vs_id).addBands(s_id)

.addBands(m_id).addBands(l_id).addBands(core)

.addBands(edge)

.rename(['id','nPixels','fCover','pSize_ha',

'vs_ha','s_ha','m_ha','l_ha','vs_id',

's_id','m_id','l_id','core','edge']);

});

## // Mapping the reducers through years

var loopsteps = ee.List.sequence(1985, 2020, 1);

var patchMetricsOutput = ee.FeatureCollection(loopsteps.map(function (x) {

### // Convert to ee.Feature

return ee.Feature(null,{

### // Adding the year as a property

'0_year': ee.Date.fromYMD(ee.Number(x), 1, 1),

### // Adding the area of very small forests as a property

'11_vs_ha': patchMetrics.filter(ee.Filter.eq('system:time_start', ee.Date.fromYMD(ee.Number(x), 1, 1)))

.toBands().rename(['id','nPixels','fCover','pSize_ha','vs_ha','s_ha','m_ha','l_ha',

'vs_id','s_id','m_id','l_id','core','edge'])

.select('vs_ha').multiply(0.09).reduceRegion({

reducer: ee.Reducer.sum(),

geometry: ROI,

maxPixels: 1e13,

scale: 30

}),

### // Adding the area of small forests as a property

'12_s_ha': patchMetrics.filter(ee.Filter.eq('system:time_start', ee.Date.fromYMD(ee.Number(x), 1, 1)))

.toBands().rename(['id','nPixels','fCover','pSize_ha','vs_ha','s_ha','m_ha','l_ha',

'vs_id','s_id','m_id','l_id','core','edge'])

.select('s_ha').multiply(0.09).reduceRegion({

reducer: ee.Reducer.sum(),

geometry: ROI,

maxPixels: 1e13,

scale: 30

}),

### // Adding the area of medium forests as a property

'13_m_ha': patchMetrics.filter(ee.Filter.eq('system:time_start', ee.Date.fromYMD(ee.Number(x), 1, 1)))

.toBands().rename(['id','nPixels','fCover','pSize_ha','vs_ha','s_ha','m_ha','l_ha',

'vs_id','s_id','m_id','l_id','core','edge'])

.select('m_ha').multiply(0.09).reduceRegion({

reducer: ee.Reducer.sum(),

geometry: ROI,

maxPixels: 1e13,

scale: 30

}),

### // Adding the area of large forests as a property

'14_l_ha': patchMetrics.filter(ee.Filter.eq('system:time_start', ee.Date.fromYMD(ee.Number(x), 1, 1)))

.toBands().rename(['id','nPixels','fCover','pSize_ha','vs_ha','s_ha','m_ha','l_ha',

'vs_id','s_id','m_id','l_id','core','edge'])

.select('l_ha').multiply(0.09).reduceRegion({

reducer: ee.Reducer.sum(),

geometry: ROI,

maxPixels: 1e13,

scale: 30

}),

### // Adding the total area of forests as a property

'15_total_ha': patchMetrics.filter(ee.Filter.eq('system:time_start', ee.Date.fromYMD(ee.Number(x), 1, 1)))

.toBands().rename(['id','nPixels','fCover','pSize_ha','vs_ha','s_ha','m_ha','l_ha',

'vs_id','s_id','m_id','l_id','core','edge'])

.select('fCover').multiply(0.09).reduceRegion({

reducer: ee.Reducer.sum(),

geometry: ROI,

maxPixels: 1e13,

scale: 30

}),

### // Adding the number o very small fragments as a property

'21_vs_id': patchMetrics.filter(ee.Filter.eq('system:time_start', ee.Date.fromYMD(ee.Number(x), 1, 1)))

.toBands().rename(['id','nPixels','fCover','pSize_ha','vs_ha','s_ha','m_ha','l_ha',

'vs_id','s_id','m_id','l_id','core','edge'])

.select('vs_id').reduceRegion({

reducer: ee.Reducer.countDistinctNonNull(),

geometry: ROI,

maxPixels: 1e13,

scale: 30

}),

### // Adding the number of small fragments as a property

'22_s_id': patchMetrics.filter(ee.Filter.eq('system:time_start', ee.Date.fromYMD(ee.Number(x), 1, 1)))

.toBands().rename(['id','nPixels','fCover','pSize_ha','vs_ha','s_ha','m_ha','l_ha',

'vs_id','s_id','m_id','l_id','core','edge'])

.select('s_id').reduceRegion({

reducer: ee.Reducer.countDistinctNonNull(),

geometry: ROI,

maxPixels: 1e13,

scale: 30

}),

### // Adding the number of medium fragments as a property

'23_m_id': patchMetrics.filter(ee.Filter.eq('system:time_start', ee.Date.fromYMD(ee.Number(x), 1, 1)))

.toBands().rename(['id','nPixels','fCover','pSize_ha','vs_ha','s_ha','m_ha','l_ha',

'vs_id','s_id','m_id','l_id','core','edge'])

.select('m_id').reduceRegion({

reducer: ee.Reducer.countDistinctNonNull(),

geometry: ROI,

maxPixels: 1e13,

scale: 30

}),

### // Adding the number of large fragments as a property

'24_l_id': patchMetrics.filter(ee.Filter.eq('system:time_start', ee.Date.fromYMD(ee.Number(x), 1, 1)))

.toBands().rename(['id','nPixels','fCover','pSize_ha','vs_ha','s_ha','m_ha','l_ha',

'vs_id','s_id','m_id','l_id','core','edge'])

.select('l_id').reduceRegion({

reducer: ee.Reducer.countDistinctNonNull(),

geometry: ROI,

maxPixels: 1e13,

scale: 30

}),

### // Adding the total number of fragments as a property

'25_total_id': patchMetrics.filter(ee.Filter.eq('system:time_start', ee.Date.fromYMD(ee.Number(x), 1, 1)))

.toBands().rename(['id','nPixels','fCover','pSize_ha','vs_ha','s_ha','m_ha','l_ha',

'vs_id','s_id','m_id','l_id','core','edge'])

.select('id').reduceRegion({

reducer: ee.Reducer.countDistinctNonNull(),

geometry: ROI,

maxPixels: 1e13,

scale: 30

}),

### // Adding the area of the largest fragment as a property

'3_largFrag_ha': patchMetrics.filter(ee.Filter.eq('system:time_start', ee.Date.fromYMD(ee.Number(x), 1, 1)))

.toBands().rename(['id','nPixels','fCover','pSize_ha','vs_ha','s_ha','m_ha','l_ha',

'vs_id','s_id','m_id','l_id','core','edge'])

.select('nPixels').multiply(0.09).reduceRegion({

reducer: ee.Reducer.max(),

geometry: ROI,

maxPixels: 1e13,

scale: 30

}),

### // Adding the mean fragment area as a property

'4_meanFrag_ha': patchMetrics.filter(ee.Filter.eq('system:time_start', ee.Date.fromYMD(ee.Number(x), 1, 1)))

.toBands().rename(['id','nPixels','fCover','pSize_ha','vs_ha','s_ha','m_ha','l_ha',

'vs_id','s_id','m_id','l_id','core','edge'])

.select('id').toInt().addBands(

patchMetrics.filter(ee.Filter.eq('system:time_start', ee.Date.fromYMD(ee.Number(x), 1, 1)))

.toBands().rename(['id','nPixels','fCover','pSize_ha','vs_ha','s_ha','m_ha','l_ha',

'vs_id','s_id','m_id','l_id','core','edge'])

.select('nPixels').multiply(0.09)).stratifiedSample({

numPoints: 1,

classBand: 'id',

region: ROI,

scale:30,

dropNulls: true

}).reduceColumns({reducer: ee.Reducer.mean(), selectors:['nPixels']

}),

### // Adding the total core area as a property

'5_core_ha': patchMetrics.filter(ee.Filter.eq('system:time_start', ee.Date.fromYMD(ee.Number(x), 1, 1)))

.toBands().rename(['id','nPixels','fCover','pSize_ha','vs_ha','s_ha','m_ha','l_ha',

'vs_id','s_id','m_id','l_id','core','edge'])

.select('core').multiply(0.09).reduceRegion({

reducer: ee.Reducer.sum(),

geometry: ROI,

maxPixels: 1e13,

scale: 30

}),

### // Adding the total edge area as a property

'6_edge_ha': patchMetrics.filter(ee.Filter.eq('system:time_start', ee.Date.fromYMD(ee.Number(x), 1, 1)))

.toBands().rename(['id','nPixels','fCover','pSize_ha','vs_ha','s_ha','m_ha','l_ha',

'vs_id','s_id','m_id','l_id','core','edge'])

.select('edge').multiply(0.09).reduceRegion({

reducer: ee.Reducer.sum(),

geometry: ROI,

maxPixels: 1e13,

scale: 30

})

});

}));

## /*********************************************************************************************/

## /******************************* Deforestation and Forest Regeneration ******************************/

## /*********************************************************************************************/

## // Generating bands for calculaling deforestation and forest regeneration through GEE reducers

var loopsteps = ee.List.sequence(1987, 2017, 1);

var defForRest = ee.ImageCollection.fromImages(loopsteps.map(function(x){

### // Setting dates for moving window

var firstYear = ee.Date.fromYMD(ee.Number(x).subtract(2), 1, 1);

var secondYear = ee.Date.fromYMD(ee.Number(x).subtract(1), 1, 1);

var thirdYear = ee.Date.fromYMD(ee.Number(x), 1, 1);

var fourthYear = ee.Date.fromYMD(ee.Number(x).add(1), 1, 1);

var fifthYear = ee.Date.fromYMD(ee.Number(x).add(2), 1, 1);

var sixthYear = ee.Date.fromYMD(ee.Number(x).add(3), 1, 1);

### // Selecting images using dates

var firstImage = myCollection.filter(ee.Filter.eq("system:time_start", firstYear))

.toBands().rename(['id','nPixels','fCover','pSize_ha']).select('fCover');

var secondImage = myCollection.filter(ee.Filter.eq("system:time_start", secondYear))

.toBands().rename(['id','nPixels','fCover','pSize_ha']).select('fCover');

var thirdImage = myCollection.filter(ee.Filter.eq("system:time_start", thirdYear))

.toBands().rename(['id','nPixels','fCover','pSize_ha']).select('fCover');

var fourthImage = myCollection.filter(ee.Filter.eq("system:time_start", fourthYear))

.toBands().rename(['id','nPixels','fCover','pSize_ha']).select('fCover');

var fifthImage = myCollection.filter(ee.Filter.eq("system:time_start", fifthYear))

.toBands().rename(['id','nPixels','fCover','pSize_ha']).select('fCover');

var sixthImage = myCollection.filter(ee.Filter.eq("system:time_start", sixthYear))

.toBands().rename(['id','nPixels','fCover','pSize_ha']).select('fCover');

### // Remapping

firstImage = firstImage.where(firstImage, 100000).unmask(200000).clip(ROI);

secondImage = secondImage.where(secondImage, 10000).unmask(20000).clip(ROI);

thirdImage = thirdImage.where(thirdImage, 1000).unmask(2000).clip(ROI);

fourthImage = fourthImage.where(fourthImage, 100).unmask(200).clip(ROI);

fifthImage = fifthImage.where(fifthImage, 10).unmask(20).clip(ROI);

sixthImage = sixthImage.where(sixthImage, 1).unmask(2).clip(ROI);

### // Summing and reclassifying

var aff = firstImage.add(secondImage).add(thirdImage).add(fourthImage).add(fifthImage).add(sixthImage);

aff = aff.updateMask(aff.eq(221111));

aff = aff.where(aff, 1); // in Ha

var def = firstImage.add(secondImage).add(thirdImage).add(fourthImage);

def = def.updateMask(def.eq(112200));

var defYear = def.where(def, ee.Number(x)); // in Ha

def = def.where(def, 1); // in Ha

### // Deforestation and fragment size

var first = myCollection.filter(ee.Filter.eq("system:time_start", firstYear))

.toBands().rename(['id','nPixels','fCover','pSize_ha']).select('pSize_ha');

var second = myCollection.filter(ee.Filter.eq("system:time_start", secondYear))

.toBands().rename(['id','nPixels','fCover','pSize_ha']).select('pSize_ha');

var def_fragSize = ee.ImageCollection([first, second]).mean().updateMask(def.where(def, 1));

var vs_def = def_fragSize.updateMask(def_fragSize.lt(10));

var s_def = def_fragSize.updateMask(def_fragSize.gte(10).and(def_fragSize.lt(100)));

var m_def = def_fragSize.updateMask(def_fragSize.gte(100).and(def_fragSize.lt(1000)));

var l_def = def_fragSize.updateMask(def_fragSize.gte(1000));

vs_def = vs_def.where(vs_def, 1);

s_def = s_def.where(s_def, 1);

m_def = m_def.where(m_def, 1);

l_def = l_def.where(l_def, 1);

### // Merging bands

return def.addBands(defYear).addBands(aff).addBands(vs_def).addBands(s_def).addBands(m_def).addBands(l_def)

.set('system:time_start', thirdYear).rename(['deforestation', 'defYear', 'fRestoration',

'defVS', 'defS', 'defM', 'defL']);

})

);

## // Applying the reducers

var defForRegOutput = ee.FeatureCollection(loopsteps.map(function (x) {

### // Convert to ee.Feature

return ee.Feature(null,{

### // Adding the year as a property

'0_year': ee.Date.fromYMD(ee.Number(x), 1, 1),

### // Adding the deforestation of very small forests as a property

'11_defVS': defForRest.filter(ee.Filter.eq('system:time_start', ee.Date.fromYMD(ee.Number(x), 1, 1)))

.toBands().rename(['deforestation','defYear','fRegeneration','defVS','defS','defM','defL'])

.select('defVS').multiply(0.09).reduceRegion({

reducer: ee.Reducer.sum(),

geometry: ROI,

maxPixels: 1e13,

scale: 30

}),

### // Adding the deforestation of small forests as a property

'12_defS': defForRest.filter(ee.Filter.eq('system:time_start', ee.Date.fromYMD(ee.Number(x), 1, 1)))

.toBands().rename(['deforestation','defYear','fRegeneration','defVS','defS','defM','defL'])

.select('defS').multiply(0.09).reduceRegion({

reducer: ee.Reducer.sum(),

geometry: ROI,

maxPixels: 1e13,

scale: 30

}),

### // Adding the deforestation of medium forests as a property

'13_defVS': defForRest.filter(ee.Filter.eq('system:time_start', ee.Date.fromYMD(ee.Number(x), 1, 1)))

.toBands().rename(['deforestation','defYear','fRegeneration','defVS','defS','defM','defL'])

.select('defM').multiply(0.09).reduceRegion({

reducer: ee.Reducer.sum(),

geometry: ROI,

maxPixels: 1e13,

scale: 30

}),

### // Adding the deforestation of large forests as a property

'14_defL': defForRest.filter(ee.Filter.eq('system:time_start', ee.Date.fromYMD(ee.Number(x), 1, 1)))

.toBands().rename(['deforestation','defYear','fRegeneration','defVS','defS','defM','defL'])

.select('defL').multiply(0.09).reduceRegion({

reducer: ee.Reducer.sum(),

geometry: ROI,

maxPixels: 1e13,

scale: 30

}),

### // Adding the deforestation as a property

'15_def': defForRest.filter(ee.Filter.eq('system:time_start', ee.Date.fromYMD(ee.Number(x), 1, 1)))

.toBands().rename(['deforestation','defYear','fRegeneration','defVS','defS','defM','defL'])

.select('deforestation').multiply(0.09).reduceRegion({

reducer: ee.Reducer.sum(),

geometry: ROI,

maxPixels: 1e13,

scale: 30

}),

### // Adding the forest regeneration as a property

'2_fRegeneration': defForRest.filter(ee.Filter.eq('system:time_start', ee.Date.fromYMD(ee.Number(x), 1, 1)))

.toBands().rename(['deforestation','defYear','fRegeneration','defVS','defS','defM','defL'])

.select('fRegeneration').multiply(0.09).reduceRegion({

reducer: ee.Reducer.sum(),

geometry: ROI,

maxPixels: 1e13,

scale: 30

})

});

}));

## /*********************************************************************************************/

## /********************************* Current Older and Younger Forests *******************************/

## /*********************************************************************************************/

## // Selecting the first deforestation event for each pixel

var firstDef = defForReg.select('defYear').min(); // first event of deforestation

## // Selecting image from the first year of your time series

var olderForests = myCollection.filter(ee.Filter.eq("system:time_start", ee.Date.fromYMD(ee.Number(1985), 1, 1)))

.toBands().rename(['id','nPixels','fCover','pSize_ha']).select('fCover');

## // Selecting older forests (pixels that were forests in the first year of your time series with no deforestation)

var notDef = firstDef.where(firstDef, 1).unmask().clip(ROI);

notDef = notDef.updateMask(notDef.eq(0));

olderForests = olderForests.updateMask(notDef.eq(0));

## /*********************************************************************************************/

## /***************************** Deforestation of Older and Younger Forests ****************************/

## /*********************************************************************************************/

var loopsteps = ee.List.sequence(1987, 2017, 1);

## // Applying reducers for calculaling deforestation of older and younger forests

var oldYoung = ee.FeatureCollection(loopsteps.map(function (x) {

### // Selecting older forests

var oldf = myCollection.filter(ee.Filter.eq("system:time_start", ee.Date.fromYMD(ee.Number(1985), 1, 1)))

.toBands().rename(['id','nPixels','fCover','pSize_ha']).select('fCover');

var mask2 = firstDef.updateMask(firstDef.lt(ee.Number(x))).unmask().clip(ROI);

mask2 = mask2.updateMask(mask2.eq(0)).add(1);

oldf = oldf.updateMask(mask2);

### // Selecting younger forests

var youngf = myCollection.filter(ee.Filter.eq("system:time_start", ee.Date.fromYMD(ee.Number(x).subtract(1), 1, 1)))

.toBands().rename(['id','nPixels','fCover','pSize_ha']).select('fCover');

var mask3 = oldf.unmask().clip(ROI).updateMask(oldf.unmask().clip(ROI).eq(0)).add(1);

youngf = youngf.updateMask(mask3);

### // Calculating deforestation of older forests

var defold = defForReg.filter(ee.Filter.eq("system:time_start", ee.Date.fromYMD(ee.Number(x), 1, 1)))

.toBands().rename(['deforestation','defYear','fRestoration','defVS','defS','defM','defL']).select('deforestation');

defold = defold.updateMask(oldf);

### // Calculating deforestation of younger forests

var defyoung = defForReg.filter(ee.Filter.eq("system:time_start", ee.Date.fromYMD(ee.Number(x), 1, 1)))

.toBands().rename(['deforestation','defYear','fRestoration','defVS','defS','defM','defL']).select('deforestation');

defyoung = defyoung.updateMask(defold.unmask().clip(ROI).updateMask(defold.unmask().clip(ROI).eq(0)).add(1));

### // Returning ee.Feature

return ee.Feature(null,{

'0_year': ee.Date.fromYMD(ee.Number(x), 1, 1),

'1_def_old': defold.multiply(0.09)

.reduceRegion({reducer: ee.Reducer.sum(), geometry: ROI,

maxPixels: 1e13, scale: 30}),

'2_def_young': defyoung.multiply(0.09)

.reduceRegion({reducer: ee.Reducer.sum(), geometry: ROI,

maxPixels: 1e13, scale: 30})

});

}));

## /*********************************************************************************************/

## /***************************************** Zonal statistics ***************************************/

## /*********************************************************************************************/

## // Area

var zonal_ha = patchMetrics.filter(ee.Filter.eq("system:time_start", ee.Date.fromYMD(2020, 1, 1)))

.toBands().select(['35_vs_ha','35_s_ha','35_m_ha','35_l_ha','35_fCover'])

.addBands(olderForests).addBands(youngerForests)

.addBands(myCollection3.select('deforestation').sum())

.addBands(myCollection3.select('fRestoration').sum())

.multiply(0.09)

.rename(['11_vs_ha_2020','12_s_ha_2020','13_m_ha_2020','14_l_ha_2020','15_total_ha_2020',

'21_old_ha_2017','22_young_ha_2017','31_def_accum_2017','32_fRest_accum_2017']);

var zonalStats_ha = zonal_ha

.reduceRegions({

collection: features,

reducer: ee.Reducer.sum(),

scale: 30

});

## // Number of fragments

var zonal_id = patchMetrics.filter(ee.Filter.eq("system:time_start", ee.Date.fromYMD(2020, 1, 1)))

.toBands().select(['35_vs_id','35_s_id','35_m_id','35_l_id','35_id'])

.rename(['vs_id_2020','s_id_2020','m_id_2020','l_id_2020','total_id_2020']);

var zonalStats_id = zonal_id

.reduceRegions({

collection: features,

reducer: ee.Reducer.countDistinctNonNull(),

scale: 30

});

## /*********************************************************************************************/

## /******************************** Identification of the Largest Fragments *****************************/

## /*********************************************************************************************/

## // Selecting fragments larger than 5000 ha

var list = patchMetrics.filter(ee.Filter.eq("system:time_start", ee.Date.fromYMD(2017, 1, 1)))

.toBands().select('32_id')

.updateMask(patchMetrics.filter(ee.Filter.eq("system:time_start", ee.Date.fromYMD(2017, 1, 1)))

.toBands().select('32_pSize_ha').gte(5000)).reduceRegion({

reducer: ee.Reducer.frequencyHistogram(),

geometry: ROI,

maxPixels: 1e13,

scale: 30

});

## // Manually check for fragments' ID [using print(list), it should be the left column values] and add it to the line below

list = ee.List([17378, 38316, 40142, 79332]);

## // Map to calculate metrics for the largest fragments

var largerFrags = ee.FeatureCollection(list.map(function (x) {

return ee.Feature(null,{

### // Adding the ID as a property

'0_id': ee.Number(x),

### // Adding the area as a property

'1_size_ha': patchMetrics.filter(ee.Filter.eq("system:time_start", ee.Date.fromYMD(2017, 1, 1)))

.toBands().select('32_fCover')

.updateMask(patchMetrics.filter(ee.Filter.eq("system:time_start", ee.Date.fromYMD(2017, 1, 1)))

.toBands().select('32_id').eq(ee.Number(x))).multiply(0.09).reduceRegion({

reducer: ee.Reducer.sum(),

geometry: ROI,

maxPixels: 1e13,

scale: 30

}),

### // Adding the older forests area as a property

'2_old_ha': olderForests.updateMask(patchMetrics.filter(ee.Filter.eq("system:time_start", ee.Date.fromYMD(2017, 1, 1)))

.toBands().select('32_id').eq(ee.Number(x))).multiply(0.09).reduceRegion({

reducer: ee.Reducer.sum(),

geometry: ROI,

maxPixels: 1e13,

scale: 30

}),

### // Adding the younger forests area as a property

'3_young_ha': youngerForests.updateMask(patchMetrics.filter(ee.Filter.eq("system:time_start", ee.Date.fromYMD(2017, 1, 1)))

.toBands().select('32_id').eq(ee.Number(x))).multiply(0.09).reduceRegion({

reducer: ee.Reducer.sum(),

geometry: ROI,

maxPixels: 1e13,

scale: 30

}),

### // Adding the core area as a property

'4_core_ha': patchMetrics.filter(ee.Filter.eq("system:time_start", ee.Date.fromYMD(2017, 1, 1)))

.toBands().select('32_core')

.updateMask(patchMetrics.filter(ee.Filter.eq("system:time_start", ee.Date.fromYMD(2017, 1, 1)))

.toBands().select('32_id').eq(ee.Number(x))).multiply(0.09).reduceRegion({

reducer: ee.Reducer.sum(),

geometry: ROI,

maxPixels: 1e13,

scale: 30

}),

### // Adding the edge area as a property

'5_edge_ha': patchMetrics.filter(ee.Filter.eq("system:time_start", ee.Date.fromYMD(2017, 1, 1)))

.toBands().select('32_edge')

.updateMask(patchMetrics.filter(ee.Filter.eq("system:time_start", ee.Date.fromYMD(2017, 1, 1)))

.toBands().select('32_id').eq(ee.Number(x))).multiply(0.09).reduceRegion({

reducer: ee.Reducer.sum(),

geometry: ROI,

maxPixels: 1e13,

scale: 30

})

});

}));

## /*********************************************************************************************/

## /***************************************** Habitat quality ***************************************/

## /*********************************************************************************************/

## // Selecting images

var core2017Reclass = img2017.select('32_core').unmask().clip(ROI);

var edge2017Reclass = img2017.select('32_edge').unmask().clip(ROI);

var old2017Reclass = olderForests.unmask().clip(ROI);

var young2017Reclass = youngerForests.unmask().clip(ROI);

## // New values according to patch size (1 - 4)

var patchSizeReclass = img2017.select('32_vs_ha').where(img2017.select('32_vs_ha'), 1).unmask(0).clip(ROI).add(

img2017.select('32_s_ha').where(img2017.select('32_s_ha'), 2).unmask(0).clip(ROI)).add(

img2017.select('32_m_ha').where(img2017.select('32_m_ha'), 3).unmask(0).clip(ROI)).add(

img2017.select('32_l_ha').where(img2017.select('32_l_ha'), 4).unmask(0).clip(ROI));

patchSizeReclass = patchSizeReclass.updateMask(patchSizeReclass.gt(0));

## // New values according to core and edge (10 and 20)

var edgeReclass = edge2017Reclass.where(edge2017Reclass, 10).unmask(0).clip(ROI);

var coreReclass = core2017Reclass.where(core2017Reclass, 20).unmask(0).clip(ROI);

var coreEdgeReclass = coreReclass.add(edgeReclass).updateMask(coreReclass.add(edgeReclass).gt(0));

## // New values according to age (100 and 200)

var olderReclass = old2017Reclass.where(old2017Reclass, 200).unmask(0).clip(ROI);

var youngerReclass = young2017Reclass.where(young2017Reclass, 100).unmask(0).clip(ROI);

var oldYoungReclass = olderReclass.add(youngerReclass).updateMask(olderReclass.add(youngerReclass).gt(0));

## // Generating habitat quality layer

var habQuality = patchSizeReclass.add(coreEdgeReclass).add(oldYoungReclass);

## /*********************************************************************************************/

## /******************************************* Exporting *****************************************/

## /*********************************************************************************************/

## // Run the batch downloads one at a time

/*

var batch = require('users/fitoprincipe/geetools:batch');

batch.Download.ImageCollection.toDrive(patchMetrics.map(function(image){

return ee.Image([]).addBands(image.select('id')).toInt().clip(ROI).copyProperties({source: image, exclude: ['count']})}),

'yourgoogledrivefoldername', {

name: 'id_{id}',

scale: 30,

region: ROI,

maxPixels: 1e13,

skipEmptyTiles: true,

crs: 'EPSG:4326',

fileFormat: "GeoTIFF"

});

*/

/*

var batch = require('users/fitoprincipe/geetools:batch');

batch.Download.ImageCollection.toDrive(patchMetrics.map(function(image){

return ee.Image([]).addBands(image.select('pSize_ha')).toFloat().clip(ROI).copyProperties({source: image, exclude: ['count']})}),

'yourgoogledrivefoldername', {

name: 'pSize_{id}',

scale: 30,

region: ROI,

maxPixels: 1e13,

skipEmptyTiles: true,

crs: 'EPSG:4326',

fileFormat: "GeoTIFF"

});

*/

/*

var batch = require('users/fitoprincipe/geetools:batch');

batch.Download.ImageCollection.toDrive(patchMetrics.map(function(image){

var edge = image.select('edge').where(image.select('edge'), 1).unmask(0).clip(ROI);

var core = image.select('core').where(image.select('core'), 2).unmask(0).clip(ROI);

var coreEdge = edge.add(core).updateMask(edge.add(core).gt(0)).rename('coreEdge');

return ee.Image([]).addBands(coreEdge.select('coreEdge')).toInt().clip(ROI).copyProperties({source: image, exclude: ['count']})}),

'yourgoogledrivefoldername', {

name: 'coreEdge_{id}',

scale: 30,

region: ROI,

maxPixels: 1e13,

skipEmptyTiles: true,

crs: 'EPSG:4326',

fileFormat: "GeoTIFF"

});

*/

/*

var batch = require('users/fitoprincipe/geetools:batch');

batch.Download.ImageCollection.toDrive(defForReg.map(function(image){

return ee.Image([]).addBands(image.select('deforestation')).toInt().clip(ROI).copyProperties({source: image, exclude: ['count']})}),

'yourgoogledrivefoldername', {

name: 'def_{id}',

scale: 30,

region: ROI,

maxPixels: 1e13,

skipEmptyTiles: true,

crs: 'EPSG:4326',

fileFormat: "GeoTIFF"

});

*/

/*

var batch = require('users/fitoprincipe/geetools:batch');

batch.Download.ImageCollection.toDrive(defForReg.map(function(image){

return ee.Image([]).addBands(image.select('fRegeneration')).toInt().clip(ROI).copyProperties({source: image, exclude: ['count']})}),

'yourgoogledrivefoldername', {

name: 'fReg_{id}',

scale: 30,

region: ROI,

maxPixels: 1e13,

skipEmptyTiles: true,

crs: 'EPSG:4326',

fileFormat: "GeoTIFF"

});

*/

Export.image.toDrive({image: olderForests.where(olderForests, 2).unmask().clip(ROI)

.add(youngerForests.unmask().clip(ROI))

.updateMask(myCollection

.filter(ee.Filter.eq("system:time_start", ee.Date.fromYMD(ee.Number(2017), 1, 1)))

.toBands()

.rename(['id','nPixels','fCover','pSize_ha'])

.select('fCover')),

description: "OldYoung_tiff",

region: ROI,

scale: 30,

maxPixels: 1e13,

fileFormat: "GeoTIFF"});

Export.image.toDrive({image: largerFrags_img,

description: "largerFrags_tiff",

region: ROI,

scale: 30,

maxPixels: 1e13,

fileFormat: "GeoTIFF"});

Export.image.toDrive({image: habQuality.float().clip(ROI),

description: "habitatQuality_tiff",

region: ROI,

scale: 30,

maxPixels: 1e13,

fileFormat: "GeoTIFF"});

Export.table.toDrive({

collection: zonalStats_ha,

description: 'zonalStats_ha_shp',

fileFormat: 'SHP'

});

Export.table.toDrive({

collection: zonalStats_id,

description: 'zonalStats_id_shp',

fileFormat: 'SHP'

});

Export.table.toDrive({

collection: patchMetricsOutput,

description: 'patchMetrics_2020_csv',

fileFormat: 'CSV'

});

Export.table.toDrive({

collection: defForRegOutput,

description: 'def_fRest_2017_csv',

fileFormat: 'CSV'

});

Export.table.toDrive({

collection: defOldYoung,

description: 'def_oldYoung_2017_csv',

fileFormat: 'CSV'

});

Export.table.toDrive({

collection: largerFrags,

description: 'largerFrags_id_csv',

fileFormat: 'CSV'

});
