# Supplementary material for: Spatiotemporal dynamics reveals forest rejuvenation, fragmentation, and edge effects in an Atlantic Forest hotspot, the Pernambuco Endemism Center, northeastern Brazil
Source: PLoS One. 2023 Sep 8;18(9):e0291234. doi: 10.1371/journal.pone.0291234 (PMC10490850; doi:10.1371/journal.pone.0291234)
Supplement: S1 Table — Class-level metrics related to forest cover area (total and by four classes of fragment size), number of fragments (total and by four classes of fragment size), largest fragment area, mean fragment area, core and edge areas. Values presented in hectares for area-related metrics. (DOCX) [file pone.0291234.s008.docx]

**S1 Table. Landscape metrics of forests over the Pernambuco Endemism Center.** Class-level metrics related to forest cover area (total and by four classes of fragment size), number of fragments (total and by four classes of fragment size), largest fragment area, mean fragment area, core and edge areas. Values presented in hectares for area-related metrics.

| Year | FA - VS | FA – S | FA – M | FA - L | FA - T | NF - VS | NF - S | NF - M | NF - L | NF - T | LFA | MFA | CORE | EDGE |
| --- | --- | --- | --- | --- | --- | --- | --- | --- | --- | --- | --- | --- | --- | --- |
| 1985 | 144,108 | 175,729 | 160,874 | 90,951 | 571,661 | 75,263 | 6,583 | 700 | 35 | 82,581 | 15,355 | 7 | 215,229 | 356,432 |
| 1986 | 140,579 | 172,447 | 157,753 | 87,156 | 557,935 | 72,999 | 6,475 | 682 | 34 | 80,190 | 15,355 | 7 | 210,715 | 347,220 |
| 1987 | 111,996 | 147,593 | 160,419 | 94,162 | 514,169 | 57,267 | 5,483 | 650 | 39 | 63,439 | 13,098 | 8 | 210,774 | 303,395 |
| 1988 | 100,484 | 140,074 | 151,727 | 86,908 | 479,193 | 49,903 | 5,137 | 622 | 38 | 55,700 | 12,455 | 9 | 200,795 | 278,398 |
| 1989 | 95,032 | 135,296 | 144,753 | 67,068 | 442,149 | 46,733 | 4,939 | 582 | 25 | 52,279 | 12,656 | 8 | 188,889 | 253,260 |
| 1990 | 88,754 | 131,961 | 142,221 | 79,029 | 441,965 | 43,014 | 4,776 | 564 | 32 | 48,386 | 12,423 | 9 | 194,059 | 247,906 |
| 1991 | 85,762 | 128,443 | 152,925 | 72,285 | 439,414 | 41,594 | 4,671 | 591 | 27 | 46,883 | 12,932 | 9 | 194,274 | 245,139 |
| 1992 | 86,307 | 130,659 | 148,281 | 76,707 | 441,953 | 41,680 | 4,762 | 588 | 32 | 47,062 | 11,393 | 9 | 195,616 | 246,337 |
| 1993 | 85,561 | 125,436 | 139,587 | 73,652 | 424,235 | 41,535 | 4,580 | 554 | 33 | 46,702 | 11,616 | 9 | 189,416 | 234,820 |
| 1994 | 85,125 | 127,626 | 141,094 | 77,019 | 430,865 | 41,100 | 4,651 | 562 | 34 | 46,347 | 11,514 | 9 | 191,801 | 239,063 |
| 1995 | 83,832 | 128,777 | 145,140 | 72,215 | 429,964 | 40,349 | 4,718 | 573 | 29 | 45,669 | 11,564 | 9 | 191,676 | 238,289 |
| 1996 | 79,778 | 129,216 | 146,931 | 73,308 | 429,232 | 38,108 | 4,670 | 568 | 29 | 43,375 | 9,297 | 10 | 195,927 | 233,305 |
| 1997 | 78,639 | 129,225 | 146,298 | 69,173 | 423,335 | 37,118 | 4,628 | 555 | 26 | 42,327 | 9,370 | 10 | 194,503 | 228,832 |
| 1998 | 75,656 | 128,008 | 144,479 | 76,248 | 424,391 | 35,117 | 4,590 | 556 | 31 | 40,294 | 9,624 | 11 | 199,147 | 225,244 |
| 1999 | 72,846 | 128,590 | 144,609 | 81,034 | 427,078 | 33,493 | 4,591 | 560 | 32 | 38,676 | 10,650 | 11 | 203,958 | 223,120 |
| 2000 | 72,485 | 128,418 | 143,952 | 81,441 | 426,296 | 32,854 | 4,589 | 558 | 33 | 38,034 | 10,688 | 11 | 204,969 | 221,326 |
| 2001 | 69,162 | 128,153 | 144,288 | 74,559 | 416,161 | 30,975 | 4,565 | 544 | 29 | 36,113 | 11,076 | 12 | 203,943 | 212,219 |
| 2002 | 67,955 | 128,055 | 141,094 | 80,267 | 417,370 | 30,005 | 4,546 | 550 | 33 | 35,134 | 11,731 | 12 | 205,849 | 211,521 |
| 2003 | 72,822 | 131,255 | 140,351 | 82,407 | 426,834 | 32,850 | 4,670 | 551 | 33 | 38,104 | 11,780 | 11 | 206,979 | 219,856 |
| 2004 | 72,054 | 131,144 | 140,471 | 81,224 | 424,893 | 32,469 | 4,647 | 553 | 32 | 37,701 | 11,824 | 11 | 206,986 | 217,906 |
| 2005 | 70,615 | 129,561 | 140,707 | 81,869 | 422,751 | 31,615 | 4,564 | 547 | 30 | 36,756 | 12,083 | 12 | 207,256 | 215,495 |
| 2006 | 70,768 | 130,421 | 138,912 | 82,658 | 422,760 | 31,788 | 4,603 | 548 | 34 | 36,973 | 11,817 | 11 | 206,462 | 216,298 |
| 2007 | 77,998 | 134,397 | 139,492 | 84,137 | 436,023 | 36,058 | 4,805 | 566 | 35 | 41,464 | 8,628 | 11 | 205,854 | 230,170 |
| 2008 | 80,199 | 134,291 | 138,568 | 84,514 | 437,572 | 37,510 | 4,832 | 562 | 37 | 42,941 | 8,614 | 10 | 205,173 | 232,399 |
| 2009 | 80,097 | 134,866 | 139,160 | 84,792 | 438,916 | 37,330 | 4,846 | 565 | 37 | 42,778 | 8,626 | 10 | 206,055 | 232,861 |
| 2010 | 76,324 | 134,608 | 142,088 | 81,850 | 434,870 | 35,257 | 4,798 | 564 | 31 | 40,650 | 11,354 | 11 | 208,249 | 226,621 |
| 2011 | 72,318 | 131,982 | 143,012 | 87,369 | 434,682 | 32,626 | 4,699 | 591 | 36 | 37,952 | 11,612 | 11 | 213,374 | 221,308 |
| 2012 | 70,378 | 131,666 | 145,809 | 95,955 | 443,808 | 30,842 | 4,704 | 602 | 39 | 36,187 | 11,876 | 12 | 221,923 | 221,885 |
| 2013 | 76,196 | 136,001 | 151,035 | 101,490 | 464,722 | 34,061 | 4,862 | 617 | 41 | 39,581 | 12,295 | 12 | 227,841 | 236,881 |
| 2014 | 78,998 | 139,749 | 155,905 | 102,308 | 476,959 | 35,692 | 5,004 | 627 | 41 | 41,364 | 12,480 | 12 | 232,503 | 244,456 |
| 2015 | 81,381 | 142,614 | 157,958 | 109,124 | 491,078 | 37,258 | 5,087 | 630 | 44 | 43,019 | 12,569 | 11 | 238,909 | 252,169 |
| 2016 | 81,713 | 144,903 | 159,790 | 110,816 | 497,222 | 37,640 | 5,152 | 631 | 42 | 43,465 | 12,668 | 11 | 241,600 | 255,621 |
| 2017 | 80,791 | 144,677 | 160,737 | 112,488 | 498,692 | 37,183 | 5,106 | 632 | 43 | 42,964 | 12,879 | 12 | 244,036 | 254,657 |
| 2018 | 81,329 | 143,797 | 159,906 | 116,523 | 501,555 | 37,232 | 5,069 | 635 | 46 | 42,982 | 12,842 | 12 | 245,376 | 256,179 |
| 2019 | 84,941 | 147,361 | 161,459 | 125,098 | 518,859 | 39,289 | 5,194 | 638 | 48 | 45,169 | 13,845 | 11 | 252,808 | 266,051 |
| 2020 | 94,091 | 154,113 | 159,495 | 132,179 | 539,877 | 44,803 | 5,519 | 641 | 54 | 51,017 | 14,643 | 11 | 255,228 | 284,649 |

FA = forest area; NF = number of fragments; LFA = largest fragment area; MFA = mean fragment area; CORE = core area; EDGE = edge area; VS = very small fragments (< 10 ha); S = small fragments (10 – 100 ha); M = medium fragments (100 – 1,000 ha); L = large fragments (> 1,000 ha); T = total.
