# Supplementary material for: Spatiotemporal dynamics reveals forest rejuvenation, fragmentation, and edge effects in an Atlantic Forest hotspot, the Pernambuco Endemism Center, northeastern Brazil
Source: PLoS One. 2023 Sep 8;18(9):e0291234. doi: 10.1371/journal.pone.0291234 (PMC10490850; doi:10.1371/journal.pone.0291234)
Supplement: S2 Table — Deforestation was classified according to fragment size (deforestation of very small, small, medium, and large fragments) and forest age (in deforestation of older and younger forests). Values presented in hectares. (DOCX) [file pone.0291234.s009.docx]

**S2 Table. Deforestation and forest regeneration over the Pernambuco Endemism Center.** Deforestation was classified according to fragment size (deforestation of very small, small, medium, and large fragments) and forest age (in deforestation of older and younger forests). Values presented in hectares.

| Year | DEF - VS | DEF - S | DEF - M | DEF - L | DEF - O | DEF - Y | DEF - T | FREG - T |
| --- | --- | --- | --- | --- | --- | --- | --- | --- |
| 1987 | 47,878 | 29,254 | 12,128 | 5,937 | – | 0 | 95,198 | 28,564 |
| 1988 | 23,043 | 17,323 | 10,583 | 4,537 | – | 852 | 55,486 | 16,154 |
| 1989 | 15,880 | 13,076 | 12,976 | 9,664 | – | 17,157 | 51,595 | 15,442 |
| 1990 | 12,292 | 8,166 | 5,663 | 1,596 | – | 6,531 | 27,717 | 13,553 |
| 1991 | 13,281 | 9,084 | 4,817 | 1,901 | – | 8,364 | 29,083 | 15,059 |
| 1992 | 10,743 | 8,371 | 6,453 | 3,749 | – | 15,211 | 29,315 | 25,113 |
| 1993 | 11,072 | 9,767 | 10,471 | 2,770 | – | 20,632 | 34,080 | 16,941 |
| 1994 | 4,848 | 3,997 | 2,770 | 924 | – | 7,570 | 12,539 | 9,223 |
| 1995 | 9,689 | 6,034 | 3,321 | 1,208 | – | 11,955 | 20,252 | 13,526 |
| 1996 | 10,934 | 7,012 | 4,324 | 791 | – | 15,960 | 23,060 | 18,369 |
| 1997 | 9,524 | 7,733 | 5,410 | 2,922 | – | 18,573 | 25,589 | 14,137 |
| 1998 | 7,218 | 5,563 | 3,522 | 1,106 | – | 12,809 | 17,409 | 12,789 |
| 1999 | 8,968 | 6,431 | 3,595 | 1,056 | – | 14,860 | 20,050 | 15,005 |
| 2000 | 4,685 | 3,464 | 2,817 | 931 | 2,658 | 9,239 | 11,897 | 8,550 |
| 2001 | 9,090 | 7,069 | 5,997 | 2,834 | 4,704 | 20,286 | 24,990 | 15,082 |
| 2002 | 3,851 | 2,827 | 1,921 | 798 | 2,556 | 6,842 | 9,398 | 10,307 |
| 2003 | 5,590 | 4,138 | 2,719 | 1,096 | 3,526 | 10,017 | 13,543 | 10,249 |
| 2004 | 3,439 | 2,398 | 1,664 | 658 | 1,771 | 6,388 | 8,159 | 7,221 |
| 2005 | 7,156 | 4,997 | 2,406 | 965 | 2,678 | 12,846 | 15,524 | 10,636 |
| 2006 | 5,744 | 3,940 | 2,417 | 1,089 | 2,482 | 10,708 | 13,190 | 12,063 |
| 2007 | 6,947 | 5,829 | 4,240 | 1,888 | 4,409 | 14,495 | 18,905 | 22,468 |
| 2008 | 1,892 | 1,178 | 629 | 534 | 391 | 3,842 | 4,233 | 1,642 |
| 2009 | 601 | 339 | 178 | 90 | 70 | 1,138 | 1,208 | 1,503 |
| 2010 | 13,561 | 7,536 | 3,851 | 1,525 | 2,201 | 24,273 | 26,474 | 17,534 |
| 2011 | 9,040 | 5,110 | 2,791 | 771 | 1,541 | 16,171 | 17,712 | 16,550 |
| 2012 | 6,317 | 3,352 | 1,605 | 420 | 1,080 | 10,614 | 11,694 | 17,021 |
| 2013 | 2,800 | 1,869 | 1,200 | 393 | 881 | 5,381 | 6,262 | 16,226 |
| 2014 | 4,343 | 2,816 | 1,596 | 616 | 884 | 8,488 | 9,371 | 17,592 |
| 2015 | 4,579 | 3,480 | 1,755 | 675 | 753 | 9,736 | 10,489 | 16,958 |
| 2016 | 5,164 | 3,256 | 1,751 | 799 | 826 | 10,143 | 10,969 | 14,586 |
| 2017 | 7,519 | 4,876 | 2,266 | 1,032 | 846 | 14,847 | 15,693 | 13,260 |

DEF = deforestation; FREG = forest regeneration; VS = very small fragments (< 10 ha); S = small fragments (10 – 100 ha); M = medium fragments (100 – 1,000 ha); L = large fragments (> 1,000 ha); O = older forests; Y = younger forests; T = total.
