# Supplementary material for: Spatiotemporal dynamics reveals forest rejuvenation, fragmentation, and edge effects in an Atlantic Forest hotspot, the Pernambuco Endemism Center, northeastern Brazil
Source: PLoS One. 2023 Sep 8;18(9):e0291234. doi: 10.1371/journal.pone.0291234 (PMC10490850; doi:10.1371/journal.pone.0291234)
Supplement: S3 Table — Forests were classified according to fragment size (very small, small, medium, and large fragments), and metrics were calculated separately for all of them and total. Values for areas presented in hectares. (DOCX) [file pone.0291234.s010.docx]

**S3 Table. Breakpoints and linear trends of forest cover area and number of fragments of the Pernambuco Endemism Center.** Forests were classified according to fragment size (very small, small, medium, and large fragments), and metrics were calculated separately for all of them and total. Values for areas presented in hectares.

| Metric | Initial year | Breakpoint year | Estimated slope | t-value | R² |
| --- | --- | --- | --- | --- | --- |
| FA - VS | 1985 | 1988 | -15,946 ± 1,476.3 | -10.801 | 0.96998 |
| FA - VS | 1988 | 2001 | -1,864.7 ± 244.69 | -7.6207 | 0.96998 |
| FA - VS | 2001 | 2019 | 673.17 ± 163.42 | 4.1191 | 0.96998 |
| FA - VS | 2019 | 2020 | 9,150 ± 4,668.3 | 1.96 | 0.96998 |
| FA - S | 1985 | 1988 | -13,137 ± 1,086.7 | -12.089 | 0.96454 |
| FA - S | 1988 | 1993 | -1,931.4 ± 1,086.7 | -1.7774 | 0.96454 |
| FA - S | 1993 | 2012 | 391.06 ± 101.78 | 3.8424 | 0.96454 |
| FA - S | 2012 | 2020 | 2,184.3 ± 313.7 | 6.9631 | 0.96454 |
| FA - M | 1985 | 1989 | -3,826.8 ± 972.6 | -3.9346 | 0.871686 |
| FA - M | 1989 | 2008 | -427.25 ± 128.82 | -3.3166 | 0.871686 |
| FA - M | 2008 | 2020 | 2,165.2 ± 257.2 | 8.4184 | 0.871686 |
| FA - L | 1985 | 1991 | -3,634.7 ± 929.31 | -3.9112 | 0.949254 |
| FA - L | 1991 | 2010 | 671.46 ± 150.75 | 4.454 | 0.949254 |
| FA - L | 2010 | 2020 | 4,404.3 ± 428.01 | 10.29 | 0.949254 |
| FA - T | 1985 | 1989 | -33,803 ± 1,892.7 | -17.859 | 0.984042 |
| FA - T | 1989 | 2001 | -1,880.3 ± 500.51 | -3.7568 | 0.984042 |
| FA - T | 2001 | 2011 | 2,232.7 ± 772.7 | 2.8895 | 0.984042 |
| FA - T | 2011 | 2020 | 10,385 ± 658.96 | 15.759 | 0.984042 |
| NF - VS | 1985 | 1988 | -9,181.2 ± 883.64 | -10.39 | 0.96998 |
| NF - VS | 1988 | 2001 | -1,149.8 ± 146.46 | -7.8506 | 0.96998 |
| NF - VS | 2001 | 2019 | 340.62 ± 97.821 | 3.4821 | 0.96998 |
| NF - VS | 2019 | 2020 | 5,514 ± 2,794.3 | 1.9733 | 0.96998 |
| NF - S | 1985 | 1989 | -533 ± 51.946 | -10.261 | 0.947493 |
| NF - S | 1989 | 2004 | -14.072 ± 6.2994 | -2.2339 | 0.947493 |
| NF - S | 2004 | 2020 | 47.115 ± 6.2994 | 7.4792 | 0.947493 |
| NF - M | 1985 | 1989 | -29.6 ± 3.0585 | -9.6778 | 0.953366 |
| NF - M | 1989 | 2005 | -2.1321 ± 0.57801 | -3.6888 | 0.953366 |
| NF - M | 2005 | 2020 | 7.1824 ± 0.52454 | 13.693 | 0.953366 |
| NF - L | 1985 | 2004 | -0.18947 ± 0.13248 | -1.4303 | 0.775609 |
| NF - L | 2004 | 2020 | 1.1765 ± 0.15658 | 7.5134 | 0.775609 |
| NF - T | 1985 | 1988 | -9,739.4 ± 921.02 | -10.575 | 0.970093 |
| NF - T | 1988 | 2001 | -1,173.7 ± 152.66 | -7.6883 | 0.970093 |
| NF - T | 2001 | 2019 | 383.05 ± 101.96 | 3.7569 | 0.970093 |
| NF - T | 2019 | 2020 | 5,848 ± 2,912.5 | 2.0079 | 0.970093 |

FA = forest area; NF = number of fragments; VS = very small fragments (< 10 ha); S = small fragments (10 – 100 ha); M = medium fragments (100 – 1,000 ha); L = large fragments (> 1,000 ha); T = total.
